# Basic taste sensitivity, eating behaviour, food propensity and BMI of preadolescent children: How are they related?

**DOI:** 10.12688/openreseurope.14117.2

**Published:** 2022-08-01

**Authors:** Ervina Ervina, Ingunn Berget, Siv Borghild Skeie, Valérie L. Almli

**Affiliations:** 1 Department of Sensory and Consumer Sciences, Nofima, Norwegian Institute of Food, Fisheries and Aquaculture Research, Ås, 1430, Norway; 2 Department of Chemistry, Biotechnology and Food Science (KBM), The Norwegian University of Life Science, Ås, 1433, Norway; 3 Food Technology Department, Faculty of Engineering, Bina Nusantara University, Jakarta, 11480, Indonesia; 4 Department of Raw Materials and Process Optimization, Nofima, Norwegian Institute of Food, Fisheries and Aquaculture Research, Ås, 1430, Norway

**Keywords:** Detection threshold, Basic tastes, Eating behaviour, Food propensity, Dairy foods, Preadolescents, BMI

## Abstract

**Background:** Taste sensitivity has been reported to influence children’s eating behaviour and contribute to their food preferences and intake. This study aimed to investigate the associations between taste sensitivity, eating behaviour, food propensity and BMI (Body Mass Index) in preadolescents.

**Methods:** Preadolescents’ taste sensitivity was measured by detection threshold of sweetness (sucrose), sourness (citric acid), saltiness (sodium chloride), bitterness (caffeine, quinine), and umami (monosodium glutamate). In addition, the Child Eating Behaviour Questionnaire (CEBQ), the Food Propensity Questionnaire (FPQ), and the children’s body weight and height were completed by the parents. A total of 69 child-parent dyads participated (preadolescents mean age =10.9 years).

**Results:** Taste sensitivity to caffeine bitterness was significantly associated with eating behaviour in food responsiveness, emotional overeating, and desire to drink. The preadolescents who were less sensitive to caffeine bitterness had higher food responsiveness scores. Those who were less sensitive to caffeine bitterness and to sweetness had higher emotional overeating scores. In addition, preadolescents who were less sensitive to sourness and bitterness of both caffeine and quinine demonstrated to have higher scores in desire to drink. There was no association between taste sensitivity and FPQ, but significant differences were observed across preadolescents’ BMI for FPQ of dairy food items, indicating higher consumption of low-fat milk in the overweight/obese compared to the normal-weight subjects. There was no significant difference in taste sensitivity according to BMI. Preadolescents’ eating behaviour differed across BMI, demonstrating a positive association between BMI and food approach, and a negative association between BMI and food avoidance.

**Conclusions:** This study contributes to the preliminary understanding of the relationships between taste sensitivity and eating behaviour in preadolescents. the results may be used to develop effective strategies to promote healthy eating practices by considering preadolescents’ taste sensitivity.

## Introduction

Taste significantly influences children’s food preference, choice, and intake ( Boesveldt *et al.*, 2018; De Cosmi *et al.*, 2017; Nguyen *et al.*, 2015). Previous studies reported that children aged 11–13 years have different intensity perceptions of basic tastes ( Ervina *et al.*, 2020; Overberg *et al.*, 2012). The individual differences in taste sensitivity could influence food preferences and eating behaviour. For instance, people with low sensitivity to sweetness and fattiness have been reported to prefer a higher intensity of these tastes in their foods to meet their optimum liking ( Cox *et al.*, 2016; Papantoni *et al.*, 2021). In addition, low sensitivity to a basic taste could be related to Body Mass Index (BMI) ( Donaldson *et al.*, 2009; Overberg *et al.*, 2012). For example, children with low sensitivity to sweet taste will seek a higher intensity of sweetness (more sugar) which can result in a higher calorie intake and a possible increase in body weight ( Cox *et al.*, 2016; Donaldson *et al.*, 2009; Papantoni *et al.*, 2021). Moreover, obese children have been reported to have a lower sensitivity to sweet taste compared to normal-weight children ( Overberg *et al.*, 2012). On the other hand, subjects with high sensitivity to bitterness prefer food with a low concentration of this taste ( Bell & Tepper, 2006; Hartvig *et al.*, 2014), thus hindering them to consume bitter dominant foods such as vegetables ( Oellingrath *et al.*, 2013) and could contribute to the insufficiency of vegetable consumption in children. Moreover, 11-year-old children were shown to have high preferences for sugary, salty, and fatty foods ( Ervina *et al.*, 2020), which are characterized as high caloric and poorly nutritious foods ( Liem & Russell, 2019). These preferences for certain foods in children could be related to their taste sensitivity and eating behaviour.

Children’s taste sensitivity has been reported to be associated with their eating behaviour. The term of eating behaviour in this paper refers to food choice and food consumption and may influences food preference and liking ( DeCosta *et al.*, 2017). Sensitivity to sweetness and bitterness measured by detection threshold in 8-9-year-old children was demonstrated to be correlated with their food preferences and lifestyle ( Rodrigues *et al.*, 2020). Moreover, a report is suggesting that subjects with low bitterness sensitivity have a higher preference for vegetables ( Keller *et al.*, 2002; Shen *et al.*, 2016; Tepper, 2008). Taste sensitivity may also be related to frequency consumption of a certain food. A study by Vennerød *et al.* (2017) showed that pre-school children aged 4–5 years who were more sensitive to sweetness consumed sweet foods less frequently. In children aged 12–13 years a more frequent consumption of fast food was associated with decreasing sensitivity to saltiness and, as a consequence, their preference for saltier beansprout soups increased ( Kim & Lee, 2009).

Exposure to different flavours and tastes during early childhood is associated with children’s food acceptability and eating behaviour when they grow older ( Nicklaus, 2016). Frequent exposures to certain basic tastes have been reviewed to be associated with increased hedonic and intensity perceptions, and this could directly influence taste satiation ( Li *et al.*, 2020). A study by Kershaw & Mattes (2018) indicated that taste exposure was more crucial than sensory sensitivity in determining food preferences and eating behaviour. Further, children aged 4–5 years who were not sensitive to bitterness had a higher acceptance for cheese and full-fat milk compared to sensitive subjects ( Keller *et al.*, 2002). Dairy products constitute one of the main structures in Norwegian children's diet at age 9 to 13 years ( Hansen *et al.*, 2016). In addition, the dairy food category provides a diverse range of essential nutrients that are highly important for children’s growth and development ( Givens, 2020). Therefore, it is of interest to investigate the influence of children’s taste sensitivity on their exposure to dairy foods.

Understanding the relationship between taste sensitivity and eating behaviour in preadolescent children will contribute to developing appropriate strategies to promote healthy eating behaviour for this age group. This is important because preadolescence is a critical period for the development of lifelong eating habits ( Gibson *et al.*, 2012) but at the same time, children in this age group have also been reported to be selective eaters ( Houldcroft *et al.*, 2014) and they may have a risk of developing childhood obesity ( Boesveldt *et al.*, 2018; Leonie *et al.*, 2018). Good eating practices that have been built and developed in preadolescence can be sustained until adolescence and may be persistent until adulthood ( Nicklaus, 2016). To achieve healthy food preferences at this age, it is important to investigate the mechanisms and determinants of child eating behaviour including the physiological aspects such as taste sensitivity. According to Nicklaus (2020), to date, comprehensive studies regarding eating and drinking habits of preadolescent children in relation to their taste sensitivity perceptions are still limited, which suggests the need for more research to be conducted within this field.

In a previous study, Ervina *et al*. (2020) measured taste sensitivity in pre-adolescents using several methods, and identified gender differences in sensitivity to sweetness and bitterness, with girls being more sensitive to these tastes. Moreover, individual differences in sensitivity to the two bitter compounds caffeine and quinine were demonstrated. The main objective of the present work is to investigate the possible influence of taste sensitivity on eating behaviour. More specifically, children-parent dyads from the study by Ervina *et al*. (2020) are explored further to investigate the association between taste sensitivity, food propensity, BMI and eating behaviour. Dairy foods are emphasised as they are an important part of the Norwegian diet ( Hansen *et al*., 2016).

## Methods

### Ethics statement

This study has been granted approval from the Norwegian Center for Research Data (NSD) No. 715734 for the data collection, the use of data for research and publication, and data management (including data processing and storage). The study is also followed the Declaration of Helsinki, while data protection has followed the General Data Protection Regulation (GDPR). A signed informed consent (written) from both the children and parents was required to participate in the study. In addition, the children’s verbal consent was also asked at the beginning of the sensory testing. The consent was asking both for participation and the use of data for research and publication purposes.

### Participants

A total of 69 children (mean age 10.9 ± 0.2 years, 46.5% boys) and their parents (one parent per child) participated in the study. The preadolescents were recruited from the 6 ^th^ grade in two primary schools located in Ski, Nordre Follo region, in Norway ( Ervina *et al*., 2020). The schools were chosen from the neighbouring municipality of the research institute to minimise the chance of involving parents affiliated with the institute. The recruitment of the participants and data collection were conducted between August-November 2019. Originally, all the 6 ^th^ grade cohort from the two schools (118 children) were invited to the study, wherein 11 did not return the consent form, one returned the form but did not complete the test, and 37 children completed the test, but their parents did not complete the questionnaire. The preadolescents performed the basic taste sensitivity test at schools while parents completed the questionnaire regarding their child’s eating behaviour and food propensity online. The participating classes received a common reward for their participation in the study, however, all the children and parents’ participation were voluntary.

### Preadolescents’ basic taste sensitivity measurement

The preadolescents’taste sensitivity was measured using the detection threshold. The detection threshold was preferred because we wanted primarily to measure the preadolescents’ physiological ability to perceive taste differences, rather than their cognitive ability to put a name on specific tastes ( Gomez *et al*., 2004; Reed & Knaapila, 2010). The children were instructed to evaluate five different concentration levels of sucrose (sweet), citric acid (sour), sodium chloride (salty), monosodium glutamate (umami), caffeine (bitter) and quinine (bitter) dissolved in water (see Ervina *et al*. (2020) for more details on sample preparations). Both the bitter compounds of caffeine and quinine were considered in this study, because our previous results suggested that preadolescents have different intensity and liking perceptions for these two compounds ( Ervina *et al*., 2020). Moreover, bitter taste has the largest number of taste compounds and taste receptors compared to the other four basic tastes ( Briand & Salles, 2016; Meyerhof *et al*., 2010). All the taste compounds were food grade and purchased from Merck Kga, Germany. Each taste compound was distinguished by different symbols, cloud (sucrose), moon (citric acid), flower (sodium chloride), sun (caffeine), star (quinine), and leaf (umami), while the different concentrations were marked by numbers from 1 (representing the lowest concentration level) to 5 (the highest concentration level). The children did not receive any information regarding the symbols, and they did not know that each series of five cups actually carried the same taste compound, or that the cup numbers corresponded to increasing concentrations. They were only informed that they would taste samples in five series of five cups marked with symbols, that different tastes could be present in any of the cups, and that the numbers on the cups indicated the order in which they should taste the samples for each series. The use of symbols was preferred to three-digit-random codes aiming to simplify the test for the preadolescents and the panel leader to ensure correct data collection. With such a large number of cups to handle, the preadolescent may have confused codes presented and/or the panel leader may easily have made mistakes when serving the samples for each participant (36 samples in total), since the samples were served in a randomized-balanced order across subjects. Visually, it is also much simpler to make sure that each child received six clouds, six stars etc., than making sure each child received six “344” cups, six “763” cups etc, thus increasing the reliability of the protocol. Moreover, a study by ( Galler *et al*., 2020) suggests that the use of symbols are preferred to code the samples to avoid confusion in children aged 6–9 years.

The preadolescents evaluated the samples in a staircase order for each series of the taste compound, starting with the lowest concentration (level 1) to the highest concentration (level 5). The children were asked “what is the taste inside this cup?” and they had to compare the sample (inside the cup) with water as a reference. They had seven options to choose from to describe the taste of each cup: “water”, “sweet”, “sour”, “salty”, “bitter”, “umami”, in addition to the option of “I don’t know”. For any symbol series and cup, the subjects were free to choose among these seven available options according to their perceptions. Thus, they could use the same option as many times as they wanted without limitations. It was technically possible to re-taste the previous cups of the same series (same taste compound, same symbol) but they could not re-taste samples from the previous series (different symbol). To ensure this practice, subjects were instructed to discard all the cups from their table after each series, so they could not interfere with the previous tasted series.

The detection threshold was obtained as the level where the subject could start to differentiate the sample from water ( Webb *et al.*, 2015), i.e. choose other options than “water”. Note that the level in which the subjects either chose the options of “I don’t know” or wrongly answered the actual taste quality was also recorded as their detection threshold, as we expect they perceived the sample to be different from water. The taste series were evaluated in a randomized balanced order across the preadolescents. The randomization was generated by the software (EyeQuestion, version 5.0.7.7, Elst, The Netherlands). The same software was also used to record the children’s responses in an online setup using a tablet. The participants always received a reminder on their screen to rinse their mouth with water to cleanse their palate between tastings of each cup. In addition, a plain cracker was offered, and the participants were instructed on screen to eat a cracker after each time they had finished one series (i.e., after they had tasted the strongest concentration, level 5). There was a short “break” for around three minutes before the participants changed to the next taste series (change stimuli). The sensory evaluation was conducted in a game-like approach called “the taste detective” ( Ervina *et al.* (2020). The application of this game-like concept aimed to increase the participation and completion rate of the children and to create a fun and engaging test activity ( Jilani *et al.*, 2019; Laureati *et al.*, 2015).

### Questionnaires to the parents

The parents were provided a link to an online questionnaire (EyeQuestions, version 5.0.7.7, Elst, The Netherlands) that was sent to their email addresses. The online questionnaire consisted of three parts. The first part asked for information regarding the family profiles such as the parent’s education level, the person in charge of preparing meals at home (mother, father, mother and father, others, buy/take-away), frequency of eating together with the family at each mealtime (breakfast, lunch, dinner, and evening meal), and frequency of the children having snacks or sweets per week. These responses were recorded in a frequency score option of 1= “never/rarely”, 2= “1–3 times per week”, 3= “4–6 times per week”, and 4= “everyday”. In addition, parents reported the weight (in kg) and height (in cm) of their child, which was then used to calculate the child’s BMI. The second part of the questionnaire consisted of the Child Eating Behaviour Questionnaire (CEBQ), while the Food Propensity Questionnaire (FPQ) was completed in the third part. It required approximately 30–35 minutes for the parents to complete the questionnaires.

***Child Eating Behaviour Questionnaire (CEBQ).*** The CEBQ was borrowed from a study by Wardle *et al.* (2001) and includes 35 statements categorized into eight different dimensions to measure children’s eating behaviour. The dimensions consist of food responsiveness (five items), enjoyment of food (four items), emotional overeating (four items), desire to drink (three items), satiety responsiveness (five items), slowness in eating (four items), emotional undereating (four items), and food fussiness (six items). The eight domains of the CEBQ assessed two global response patterns to foods known as “food approach” (includes food responsiveness, emotional overeating, enjoyment of food, desire to drink) and “food avoidance” (satiety responsiveness, slowness in eating, emotional undereating, food fussiness) ( Vandeweghe *et al.*, 2016). The complete explanation of each dimension in CEBQ has been previously reviewed ( Freitas *et al.*, 2018). The questionnaire was translated from English to Norwegian, then back translated for validation and adjustments by the research team and colleagues at the department, in Nofima, Ås.

The parent’s responses to the CEBQ were recorded in a five-point agreement scale ranging from 1= “completely disagree”, 2= “disagree”, 3= “neither agree nor disagree”, 4= “agree”, and 5= “completely agree” ( Wardle *et al.*, 2001). The CEBQ has good reliability and validity to evaluate eating behaviour in children aged 5–6 years ( Quah *et al.*, 2019), 5–12 years ( Njardvik *et al.*, 2018), and 7–12 years ( Tay *et al.*, 2016). Moreover, the CEBQ has been applied in different countries ( Santos *et al.*, 2011; Sleddens *et al.*, 2008; Tay *et al.*, 2016), and the results indicated that CEBQ is a good instrument to evaluate eating behaviour in children ( Freitas *et al.*, 2018).

***The Food Propensity Questionnaire (FPQ).*** The FPQ was completed by the parents and aims to measure how often the children ate the selected food items (i.e., frequency of consumption). The questionnaire consisted of nine different food categories involving 81 selected food items such as 1) starchy foods (bread, pasta orrice, and potatoes) (3 items), 2) spreads, toppings, and sandwich fillings (18), 3) breakfast cereals (3), 4) dairy products (7), 5) meat, fish, seafoods, soups (13), 6) vegetables (9), 7) fruits and berries (11), 8) desserts, cake, snacks, and sweets (11), and 9) drinks (6). The total dairy products listed in the questionnaire consisted of 14 items: 5 items listed in the spread, topping and sandwich filling category (brown whey cheese, semi-hard cheese, spreadable cheese, parmesan, and butter); 7 items listed in the dairy milk category (whole milk, low-fat milk, skimmed milk, fermented milk, chocolate/strawberry-flavoured milk, plain yogurt, fruit yogurt), and 2 items listed in the dessert, sweet, cake and snacks category (ice cream and dairy pudding) (see Extended data to access the complete questionnaire). The food items were then further categorized according to their basic taste profiles into sweet (35 food items), sour (11), salty (25), bitter (10), and umami (13) foods. This classification was based on the food taste profile database developed by Martin, Visalli, Lange, Schlich, and Issanchou (2014). The food taste database consists of nearly 600 food items evaluated by trained panellists based on a SpectrumTM-like profiling approach for the five basic tastes and fattiness. Note that in our study, the foods were categorized into multiple taste categories according to the database (e.g., grapefruit was rated as both bitter (score=4.3) and sour (score=3.9) in the database, therefore this food was classified as both bitter and sour in our analyses). The parent’s responses regarding FPQ of a certain food were recorded in an eating frequency with options of “never/rarely”, “1–3 times per month”, “1–3 times per week”, “4–6 times per week”, “daily”, and “more than once a day” which further transformed into Daily Frequency Equivalence (DFE). The list of food items was presented and evaluated in a random order within categories across parents.

### Data analysis

The preadolescents’ BMI was calculated from the weight (kg) and height (cm) reported by the parents. The classification for the weight status into obesity, overweight, normal, and underweight groups followed the BMI/age chart standard for school-age children, the chart is separated for boys and girls respectively WHO (2007). However, due to the limited sample size, children with underweight and normal BMI were merged into the group “normal”, whereas the overweight and obese subjects were merged into an overweight/obese group. The differences for gender and BMI status were evaluated using chi-square analysis. Parent’s education, responsible person for preparing meals at home, frequency of eating together in the family, and frequency of eating snacks or sweets of the children were analysed descriptively.

***Child Eating Behaviour Questionnaire (CEBQ).*** The association between taste sensitivity and eating behaviour was investigated using linear regression with the CEBQ score as the response variable and detection threshold of the six different taste compounds employed as the explanatory variables. The models were computed for each CEBQ domain and each taste compound separately (eight CEBQ domains and six taste compounds). A Bonferroni test was carried out for multiple testing correction. Residual distribution and data variance was checked in the analysis. The BMI groups for each domain of the eating behaviour were compared using a Mann-Whitney test. In addition, Principal Component Analysis (PCA) was applied to map the associations between CEBQ, taste detection threshold, and z-BMI score. The PCA was computed with children as rows and CEBQ (per domain) as columns, while BMI and detection threshold were involved as supplementary variables.

***The Food Propensity Questionnaire (FPQ).*** The FPQ score of eating frequency for each food item was converted into Daily Frequency Equivalence (DFE) following a study by Laureati *et al.* (2020). The score was computed by converting the eating frequency scale proportionally into one equivalence a day (the score of DFE= 1, meaning that the food item was consumed daily). The DFE score for eating frequency in the present study became as follows: DFE 0 = never/rarely, 0.07 = 1–3 times/month, 0.25 = 1–3 times/week, 1 = daily, 2 = more than once a day.

The general relationship between taste sensitivity and food propensity was analysed using a mixed model ANOVA with DFE as the response variable with the detection threshold level and the taste qualities (sweet, sour, salty, bitter-caffeine, and umami) were employed as explanatory variables. In this model, the interaction between the detection threshold and taste quality was included and child was involved as random effect. The restricted maximum likelihood (REML) method was applied for fitting the model.

To further investigate the effect of taste sensitivity on FPQ per taste (i.e., sweet foods, sour foods, salty foods, etc.), five linear regression models were computed with taste detection threshold as explanatory variable and FPQ scores (each basic taste) as response variables. The detection threshold of caffeine was chosen to represent the bitterness sensitivity as this compound has more commonly been used in taste sensitivity and dietary studies than quinine ( Ahrens, 2015; James *et al.*, 1997; Puputti *et al.*, 2019; Rodrigues *et al.*, 2020). Moreover, caffeine is used as the standard for measuring the bitterness sensitivity according to the international standardisation organization, ISO (2011).

The association between FPQ of dairy products and taste sensitivity was also evaluated using mixed model ANOVA. One model for each basic taste was applied with the FPQ score as response variable, food item, the detection threshold and BMI group were involved as explanatory variables, and child within BMI group was included as a random effect. The significant differences across different BMI groups for each dairy food item were further computed using a Mann-Whitney test. The association between FPQ of dairy products and taste sensitivity was also evaluated using mixed model ANOVA. One model for each basic taste was applied with the FPQ score as response variable, food item, the detection threshold and BMI group were involved as explanatory variables, and child within BMI group was included as a random effect. The significant differences across different BMI groups for each dairy food item were further computed using a Mann-Whitney test. The association between taste sensitivity and BMI was investigated using linear regression models. The models were computed separately for each taste compound (sweet, sour, salty, bitter-caffeine, bitter-quinine, umami) as explanatory variables, BMI score was employed as a response variable. All data were analysed using the XLSTAT Sensory version 2020.3.1 (Addinsoft, Paris, France). Power analysis using the software developed by Faul, Erdfelder, Buchner, and Lang (2009) shows that with 69 subjects the experiment has good statistical power (0.8) with medium effect size.

## Results

### Family eating habits and preadolescents BMI distribution

Most of the parents who participated in this study have a university degree (at least Bachelor’s) as their highest level of education (79%). Mothers were most frequently responsible for cooking at home (55%), while fathers alone accounted for only 10%. Shared meal responsibility (mother and father) applied in 33% of the homes. Most of the children ate dinner together with their parents almost every day (93%), quite often for breakfast (46%) but less often for lunch (10%). In addition, more than 90% of the parents gave their child snacks or sweets 1–3 times per week.

Based on the computed BMI ( Table 1), most of the children were categorized to have a normal weight status (68%, n=47,), followed by overweight (26%, n=18, 50%), obese (4%, n=3), and one child was categorized as underweight (1%, n=1). The gender distribution wasbalanced across the BMI groups (p-value > 0.05 based on chi-square analysis).

**Table 1.** Subjects’ BMI and age (N=69).

Variables		Boys	Girls	Total
BMI Status *	Underweight	0%	1% (1)	1% (1)
	Normal weight	32% (22)	36% (25)	68% (47)
	Overweight	13% (9)	13% (9)	26% (18)
	Obese	1% (1)	3% (2)	4% (3)
Mean age		(10.9 ± 0.2)	(10.9 ± 0.3)	(10.9 ± 0.2)

*BMI status was calculated based on z-score BMI ( WHO, 2007)

### Children’s taste sensitivity and eating behaviour

**Figure 1.**
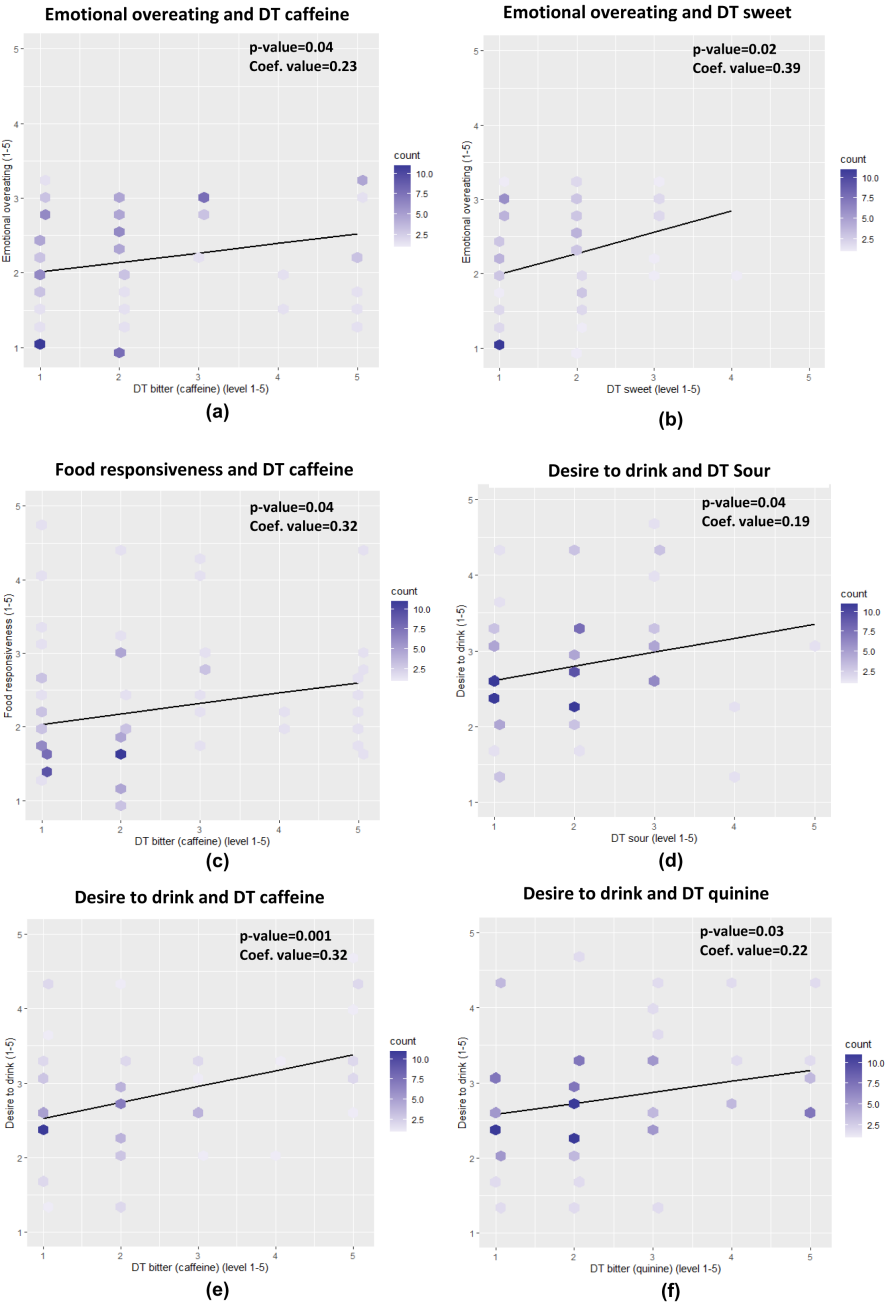
The associations between children’s taste sensitivity and eating behaviour for emotional overeating ( **a**, **b**), food responsiveness ( **c**), and desire to drink ( **d**, **e**, **f**) (DT= detection threshold, count= number of children). A more saturated colour indicates more children with the same response score Relationships between eating behaviour (CEBQ), food propensity (FPQ), and weight status (BMI).

The Cronbach's alpha of CEBQ showed a good internal consistency for both food approach and food avoidance (Cronbach's alpha = 0.84 and 0.75, respectively) and each of the CEBQ domains also showed a good internal consistency (all Cronbach's alpha ≥ 0.75) except for food fussiness and desire to drink (0.63 and 0.66, respectively). The preadolescents’ taste detection threshold was positively associated with some aspects of their eating behaviour, the effect size was, however, small with regression coefficients in the range 0.19 to 0.39 ( Figure 1). A significant and positive association was found between food responsiveness and threshold for bitter caffeine (p=0.04). The preadolescents who were less sensitive (higher detection threshold), to caffeine bitterness had a higher food responsiveness score which indicates that these subjects tend to overeat. In addition, preadolescents less sensitive to sweetness (p=0.02) and caffeine bitterness (p=0.04), also have a significantly higher score for emotional overeating. Significant and positive associations were also found in sourness (p=0.04), bitterness of caffeine (p<0.01) and quinine (p=0.03) towards the desire to drink, demonstrating that subjects who were less sensitive to these tastes have a higher score for desire to drink, which relates to the consumption of sweetened beverages.

**Figure 2.**
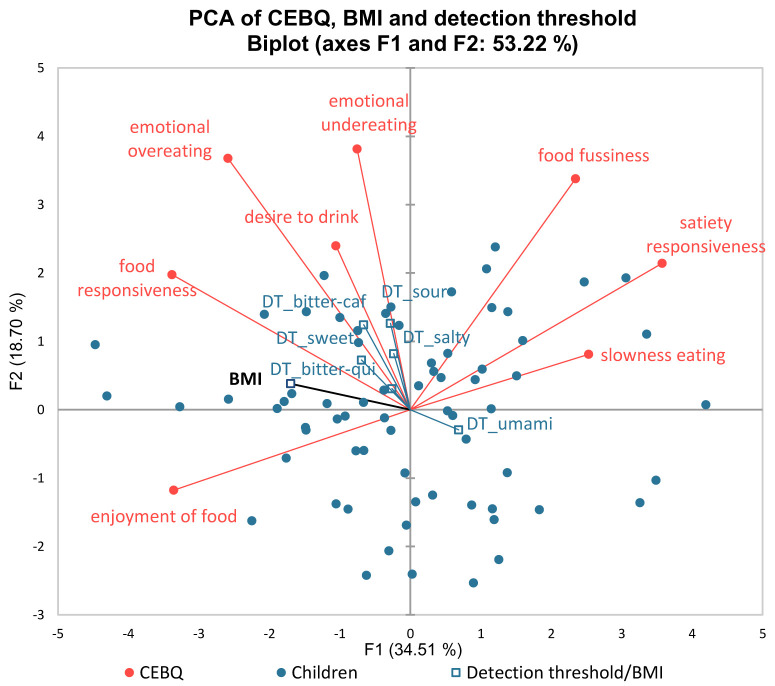
PCA biplot of CEBQ, detection thresholds, and BMI (DT=detection threshold; PCA = principal component analysis; CEBQ = Child Eating Behaviour Questionnaire; BMI = body mass index).

The PCA analysis showed that food responsiveness, emotional overeating, desire to drink, and enjoyment of food were positively associated with the children’s BMI ( Figure 2). In contrast, satiety responsiveness, slowness in eating, and food fussiness were associated with lower BMI on factor 1. The Mann-Whitney test comparing the two groups (normal and overweight/obese) for each eating behaviour domain demonstrated a significantly higher score (p≤0.05) for overweight/obese children in emotional overeating and food responsiveness. Moreover, normal weight subjects have a significantly higher score (p≤0.05) in satiety responsiveness and slowness in eating compared to the overweight/obese subjects. The PCA biplot ( Figure 2) also displays positive associations between BMI and detection thresholds, where the detection thresholds were included as supplementary variables.

**Figure 3.**
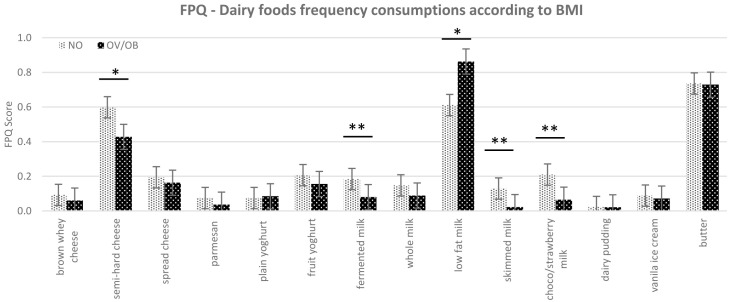
The food frequency consumption (FPQ) of dairy food items according to subjects classified as NO=normal weight and OV/OB=overweight/obese children (FPQ = Food Propensity Questionnaire; BMI = body mass index), *p≤0.1, **p≤0.05 based on mean value on Mann Whitney.

Based on linear regression, our results did not show a significant influence of taste sensitivity on children’s BMI. Neither could we detect any significant effect of children’s basic taste sensitivity on FPQ score in general, showing that taste sensitivity threshold did not relate to frequency consumption of certain foods. However, when the BMI variable was involved in the model, there were significant differences in the FPQ for the selected dairy products between the normal weight and overweight/obese children. These differences were significant for semi-hard cheese (p=0.08), fermented milk (p=0.02), skimmed milk (p=0.03), and chocolate/strawberry flavoured milk (p≤0.01), showing that normal weight subjects frequently consumed these dairy food items compared to overweight/obese ( Figure 3). Moreover, there was a tendency for the overweight/obese group to consume more low-fat milk (0.5–1.8% fat) compared to the normal weight group (p=0.1).

## Discussion

### Associations between children’s basic taste sensitivity and eating behaviour

The results showed that preadolescents who were less sensitive to caffeine bitterness have a higher food responsiveness score. Bitter taste is commonly associated with food aversion ( Reed & Knaapila, 2010; Reed *et al.*, 2006) and this taste acts as a “barrier” for humans not to ingest poisonous foods ( Mennella & Bobowski, 2015) since bitter taste is biologically linked with poisonous substances ( Reed & Knaapila, 2010). The higher food responsiveness score reflects a higher appetite and an increased desire to eat ( French *et al.*, 2012). The association between low sensitivity to bitterness and higher food responsiveness could be explained by the loosening “barrier” of bitter taste that may be triggering children to eat more food types, and therefore more food in general. A study by Goldstein *et al.* (2007) also provides a similar result to our study, showing that nine-year-old children who were less sensitive to bitterness of PROP had a higher daily energy intake compared to children who were sensitive to bitterness. This result shows an association between bitterness sensitivity and food intake that could correspond to food responsiveness in CEBQ.

Emotional overeating in CEBQ represents an increase in food intake as a response to negative emotions such as anxiety, anger, and boredom ( Freitas *et al.*, 2018; Hill *et al.*, 2018; Macht, 2008). Our results show that preadolescents who were less sensitive to sweetness and bitterness of caffeine have a higher emotional overeating score. This could increase their food intake when they experience negative emotions. Comfort foods such as sweet and fatty foods have been reported to be typically consumed in the presence of negative emotions ( Jacques *et al.*, 2019; Michels *et al.*, 2012; Pilska & Nesterowicz, 2016; van Strien *et al.*, 2013). Negative emotions can modulate desire to eat ( Macht, 2008) and subjects with emotional eating attitudes have learned to cope with their negative feelings by eating comfort foods as a way to find satisfaction ( Adam & Epel, 2007; Macht, 2008; Michels *et al.*, 2012). Michels *et al.* (2012) also conclude that eating triggered by negative emotions in children aged 5–12 years was positively correlated with their sweet food consumption. In addition, Ashi *et al.* (2017) reported that children aged 13–15 years who were less sensitive to sweetness had a significantly higher intake for sweet foods. These findings support the association between sweet taste sensitivity and emotional overeating behaviour found in our study. Further, several studies have suggested that low sensitivity to bitterness could increase children’s food intake ( Goldstein *et al.*, 2007; Keller & Adise, 2016; Tepper *et al.*, 2011). Previous studies also report that food intake in children could be modulated by negative emotions ( Hill *et al.*, 2018; Macht, 2008; Michels *et al.*, 2012). This may happen because bitter taste is “naturally” associated with poisonous substances and this taste helps humans to avoid ingestion of harmful foods ( Mennella & Bobowski, 2015). By lowering the sensitivity of bitterness, the barrier to prevent the ingestion may also be disrupted and may increase the food intake in general. This could explain the relationships found between sensitivity to bitterness and emotional overeating in this study.

Our results also show that taste sensitivity for sourness and bitterness (both caffeine and quinine) were significantly associated with desire to drink. The preadolescents who were less sensitive to these tastes have higher score for desire to drink. The CEBQ domain for desire to drink aimed to identify children’s desire for drinking, in particular for sweetened beverages, and this domain has previously been associated with food approach ( Freitas *et al.*, 2018; Quah *et al.*, 2019; Sweetman *et al.*, 2008; Vandeweghe *et al.*, 2016; Wardle *et al.*, 2001). Our result corroborates with a previous study by Mennella *et al.* (2005), which demonstrated that 5-10-year-old children who were not sensitive to PROP bitterness had heightened preferences for sweet beverages and soft drinks, and preferred more sugar added in their cereals and beverages. Interestingly, the sourness perception and thirst regulation occurred via the same acid-sensing receptor cell, which is called polycystic kidney disease 2-like 1 (PKD2L1) ( Bichet, 2018; Gravina *et al.*, 2013). A study by Zocchi *et al.* (2017) also revealed that mice without PKD2L1 showed a total loss in water and acid responses, indicating that this receptor plays an important role in both water and sourness perceptions. This suggests that the activation in the receptor cell PKD2L1 due to sourness perception could modulate desire to drink. The involvement of the same receptor in the molecular mechanism of perception could be a potential underlying reason for the significant relationship found between sourness sensitivity and desire to drink in this study.

### Caffeine and quinine sensitivities and eating behaviour

In our study, food responsiveness and emotional overeating in the preadolescents were associated with their sensitivity to caffeine bitterness but not to quinine bitterness. Preadolescent children demonstrated individual differences in the perception of different bitter compounds such as caffeine and quinine, as was reported in our previous study using data from the same participants ( Ervina *et al.*, 2020). The different bitter taste compounds have different bitterness profiles, and they elicit various intensity perceptions of bitterness ( Kamerud & Delwiche, 2007; Yokomukai *et al.*, 1993). Compared to the other four basic tastes, bitter taste has the largest number and greatest variety of compounds, with more than 25 bitterness receptors responsible for bitter taste perceptions in humans ( Meyerhof *et al.*, 2010; Roura *et al.*, 2015). Caffeine and quinine may not activate the same bitterness receptors, and this could result in differences in bitterness perception ( Kamerud & Delwiche, 2007). Moreover, these two bitter compounds are not found in the same foods ( Briand & Salles, 2016), which may also explain the different relationships between bitterness in quinine and caffeine and eating behaviour in children.

### Relationship between basic taste sensitivity and FPQ

According to our results, no significant associations were found between taste detection threshold and FPQ score for any of the five taste modalities (i.e., FPQ of sweet foods, salty foods, bitter foods, etc.). Further analyses did not show any systematic relationships between detection thresholds and food propensity for a given taste (i.e., between sweet threshold and sweet foods, salty threshold with salty foods, etc.). These results indicate that basic taste sensitivity did not systematically relate to the eating frequency of foods with the same dominant basic taste.

Parents of preadolescents still act as the primary food providers at home and have control over their child’s eating practices ( Houldcroft *et al.*, 2016). Moreover, preadolescents have been characterized as curious and autonomous eaters, but their drinking and eating habits are still framed by their parental food practices ( Nicklaus, 2020). The children’s eating frequency recorded by FPQ may not, however, capture what is children’s “actual” consumption but rather indicate what is “served” to them by their parents. FQP filled in by parents will, for instance, not reflect food that is consumed without parental supervision (i.e., eating outside home). In the USA, as many as 35% of children in early adolescence have been reported to eat outside home, with a big contribution of fast food ( Reicks *et al.*, 2015). In Norway, school lunch is typically brought from home, however, children aged 13 or more may buy food and drinks at school or in its surroundings, and most of these food choices are considered to be unhealthy ( Hilsen *et al.*, 2010) In addition, Norwegian school children aged 14-15 years buy soft drinks at school more than twice a week ( Bere *et al.*, 2008). This indicates that children may have eating activities outside home and these might not be recorded by the questionnaire. The FQP may therefore have low precision when it comes to revealing possible relations between food frequency and taste sensitivity. Differences between reports by parents and children could be of interest for follow-up studies. Further studies on relations between taste sensitivity and food propensity should both involve a more complete FPQ, as well as a larger number of subjects.

### Preadolescents’ BMI

According to the Norwegian Institute of Public Health (2018), the prevalence for overweight and obesity in nine-year-old children was recorded to be 18% and 3%, respectively. These numbers corroborate a previous study by Júlíusson *et al.* (2010) who calculated the overweight and obesity prevalence with more than 6000 Norwegian school children from 2–19 years old. Their research highlighted a higher prevalence of overweight and/or obesity in children aged 6–11 years (17%) than in younger children aged 2–5 years (12.7%) and the risk was increased with lower parental education. Our results showed a higher number for overweight (28%) compared to the above literature. This could be due to erroneous data from parents since the BMI data were not collected by direct anthropometry measurement and could also be an artefact of our small sample size or reflect regional differences.

### Association between dairy food propensity and children’s BMI

There were significant differences in the frequency consumption of dairy foods across different BMIs. The differences were significant in fermented milk, skimmed milk, and flavoured milk (chocolate/strawberry milk). Low-fat milk is the only dairy food that was consumed more frequently by the overweight/obese group, while the other dairy foods (semi-hard cheese, fermented milk, skimmed milk, and flavoured milk) were consumed more frequently by normal-weight children or consumed in nearly equal frequency between the groups (for example butter and cheese spread).

The Norwegian legislation (2015) classifies pasteurized milk into three categories according to fat content; full fat milk (≥ 3.5% fat), low-fat milk (0.5–1.8% fat), and skimmed milk (< 0.5% fat). The Norwegian Directorate of Health (2016) recommends low-fat milk for regular consumption. Milk is a standard lunch drink in Norway, and it is therefore also recommended that schools in Norway only serve drinking milk with ≤ 0.7% fat content (low-fat). This could explain the higher consumption of low-fat milk compared to whole milk or skimmed milk in our results. Moreover, the higher consumption of low-fat milk in the overweight/obese group probably appears because parents choose dairy milk products with lower fat content when they realize their child is overweight/obese, especially as almost 80% of the parents in this study had a high education level (bachelor’s degree or higher). This could also be the reason for the lower cheese and flavoured milk consumption in overweight/obese children since these products have a high fat or sugar content. Parental education level has been reported to be positively associated with diet quality of children aged 10–11 years ( Cribb *et al.*, 2011; van Ansem *et al.*, 2014). These diets are characterized by a lower intake in sugar and fat ( Fernandez-Alvira *et al.*, 2013). Moreover, in the obesogenic environment, control and monitoring of children’s food environment and intake by parents were essential to reduce children’s weight status to be close to normal weight ( Gahagan, 2012). Interestingly, a recent systematic and meta-analysis review by Vanderhout *et al.* (2020) suggested that higher consumption of whole milk is associated with low adiposity in childhood, which could be related to the results found in our study.

### Relationships between preadolescents’ basic taste sensitivity, BMI, and eating behaviour

Our results did not show a significant association between taste sensitivity and BMI in preadolescents. However, previous studies suggest that overweight and obese children have a lower taste sensitivity ( Cox *et al.*, 2016; Overberg *et al.*, 2012; Papantoni *et al.*, 2021; Rodrigues *et al.*, 2020). The reason we could not find a relationship between taste sensitivity and BMI could be due to our small data set and the unbalanced number of children between the BMI groups (70% normal weight and 30% overweight/obese). However, Cox *et al.* (2016) reported that the relationship between taste sensitivity and weight in children is still debatable and requires more evidence. A recent study investigating taste sensitivity and BMI in children aged 7–12 years found no significant differences for sweetness and bitterness sensitivity between the normal-weight and overweight groups ( Lim *et al.*, 2021). A similar result was also reported by Alexy *et al.* (2011) in a study involving a large sample set of 574 children and adolescents aged 10–17 years. Their study concluded that there was no significant difference in basic taste sensitivity across different BMIs. Factors other than taste sensitivity such as food preferences, parental feeding practices, genetic factors, obesogenic environment, and family social economic status have been reported to play significant roles in the determination of children’s BMI ( Donaldson *et al.*, 2009; Leonie *et al.*, 2018; Lytle, 2009; Scaglioni *et al.*, 2018; Woo Baidal *et al.*, 2016).

Correlations were found between children’s BMI and their eating behaviour based on the PCA mapping. The results indicated that the food approach domains such as food responsiveness, emotional overeating, desire to drink, and enjoyment of foods were positively associated with children’s BMI, while food avoidance such as satiety responsiveness, slowness in eating, and food fussiness were negatively correlated with preadolescents’ BMI. The differences between the BMI groups were confirmed with student t-tests. Our results corroborate previous studies that used the same CEBQ instrument ( Sanchez *et al.*, 2016; Santos *et al.*, 2011; Viana *et al.*, 2008; Webber *et al.*, 2009). Eating behaviour of food approach in CEBQ has previously been associated with increased BMI in children ( Braet & Van Strien, 1997; Vandeweghe *et al.*, 2016).

The PCA mapping also demonstrated that in general, a higher detection threshold (lower taste sensitivity), may relate to a higher BMI, and this might be associated with the food-approach domain of CEBQ. Children’s BMI has been reported to differ according to their taste sensitivity ( Cox *et al.*, 2016; Overberg *et al.*, 2012; Papantoni *et al.*, 2021) and children’s eating behaviour in the food approach domain was also strongly correlated with higher BMI ( Freitas *et al.*, 2018; French *et al.*, 2012; Quah *et al.*, 2019; Sanchez *et al.*, 2016). This indicates that taste sensitivity could mediate the complex interplay between eating behaviour and BMI in preadolescent children.

### Study limitations

Our study involved a limited number of participants and an unbalanced number of children for each BMI group, as a result of recruitment of whole school classes. We recommend involving more participants and to have a balanced number between normal and overweight/obese subjects for future studies. One possibility could be by involving hospitals or healthcare centres that are dealing with obesity treatment in children. Moreover, the preadolescents’ BMI was determined by a parent-reported questionnaire of body weight and height. An actual anthropometric measurement of children’s weight and height is recommended for more precise data ( Linchey *et al.*, 2019). In addition, this study is a cross-sectional design, which makes us unable to confirm the direction of the associations found in this study. We are not able to conclude whether the different frequency consumption induced overweight/obesity or whether the overweight/obesity induced the frequency consumption. Moreover, the aetiology of overweight and obesity is multifaceted, complex and involves multiple factors ( Lytle, 2009), thus, assessing the aspect only from taste sensitivity and frequency of consumption may not be enough.

Our study may also suffer from a cross-cultural issue. The FPQ was developed according to Norwegian foods, but the classification of the food according to its taste (sweet, sour, salty, bitter, umami) was conducted based on a French food database. Although all the foods listed in the FPQ are available in the database, taste profiles may be different between Norwegian and French foods.

There was a possibility of cross-modal correspondence effect in our study between taste and visual stimuli because we were using different symbols to label different taste compounds of the samples. For example, the use of a “cloud” symbol to represent sweet taste could influence children’s perception due to this cross-modal effect. Spence (2011) reported that cross-modal correspondence between different sensory modalities such as between visual and taste stimuli could influence taste perception. A previous study also reports a significant association between certain symbols and specific taste of cheeses, suggesting a moderate effect of cross-modal correspondence in sensory perceptions ( Spence *et al.*, 2013).

## Conclusion

This study aimed to investigate the association between taste sensitivity and eating behaviour in preadolescents. The results indicate a positive association between higher detection threshold (lower sensitivity) and higher scores in the food approach domain of CEBQ. There was no influence of children’s taste sensitivity on their food propensity. However, children differed according to their BMI for the propensity of dairy foods. Further, our results confirmed a positive relationship between children’s BMI and food approach, and a negative relationship between BMI and food avoidance. To our current knowledge, this is the first study to investigate the association between taste sensitivity and eating behaviour in 11-year-old children with all basic tastes (sweetness, sourness, saltiness, bitterness, umami) and with two bitter taste compounds of caffeine and quinine employed in the study.

Eating behaviour is influenced by many factors ( DeCosta *et al.*, 2017; Scaglioni *et al.*, 2018) such as parental feeding practices, family environments, parents’ education and economic condition, the obesogenic environment, and media exposure. Our results indicate that taste sensitivity also modulates eating behaviour in preadolescents. This study contributes to understanding the association between taste sensitivity and eating behaviour of preadolescent children by considering their taste sensitivity. The results could be used as preliminary findings to design future studies involving a larger number of participants as well as other cultures.

## Data availability

### Underlying data

Zenodo: Taste sensitivity and eating behaviour of preadolescent children - extended data. https://doi.org/10.5281/zenodo.5468625 ( Ervina, 2021).

This project contains the following underlying data within the file ‘Extended data taste sensitivity and eating behaviour (2).xlsx’:

- Complete data (consisted of children’s and parents’ responses, including the taste detection threshold data, parents’ education, children’s BMI, CEBQ and FPQ)
- CEBQ data (consisted of parents’ responses for each domain of CEBQ)

### Extended data

Zenodo: Taste sensitivity and eating behaviour of preadolescent children - extended data. https://doi.org/10.5281/zenodo.5468625 ( Ervina, 2021).

This project contains the following extended data within the file ‘Extended data taste sensitivity and eating behaviour (2).xlsx’:

- CEBQ_Quest (CEBQ questionnaire in English and Norwegian)
- FPQ_Quest (FPQ questionnaire in English)

Data are available under the terms of the Creative Commons Attribution 4.0 International license (CC-BY 4.0).
