# Supplementary material for: Basic taste sensitivity, eating behaviour, food propensity and BMI of preadolescent children: How are they related?
Source: Open Res Eur. 2024 Feb 2;1:127. Originally published 2021 Oct 20. [Version 3] doi: 10.12688/openreseurope.14117.3 (PMC10904958; doi:10.12688/openreseurope.14117.3)
Supplement: Peer review history

# Reviewer report: Reviewer response for version 3

**DOI:** 10.21956/openreseurope.18509.r37953

**Published:** 2024-02-29

**Authors:** Sylvie Issanchou

**Affiliations:** 1 Centre des Sciences du Goût et de l'Alimentation, CNRS, INRAE, Institut Agro, Université de Bourgogne, F-21000 Dijon, France

Authors have answered to all the comments and have made changes according to my suggestions. Table 2 is very clear and useful. I have only a few  minor comments, one of them requests verification by the authors.

 - Page 7, bottom of the left column

 In page 7 you wrote “The FPQ score of eating frequency for each food item was converted into Daily Frequency Equivalence (DFE) following a study by Laureati et al. (2020)”. Thus, in the third paragraph on The Food Propensity Questionnaire (FPQ), you should use “DFE scores” and not “FPQ scores” in the sentence “…, five linear regression models were computed with taste detection threshold as explanatory variable and FPQ scores (each basic taste) as response variables.”

 - Page 7, top of the right column

 Same comment than above for the first sentence at the top of the right column, i.e., in the sentence “One model for each basic taste was applied with the FPQ score as response variable, food item, the detection thresh[1]old and BMI group were involved as explanatory variables” you should replace “FPQ scores” by “DFE scores”

 - Page 9, bottom of the left column

 "These differences were significant for semi-hard cheese (p=0.08)": you could not say that the difference was significant with p=0.08, in particular since you performed 5 tests. You should speak about a tendency.

 - Figure 2

 I am surprised that the standard deviations bars for NObese and for OV/OB overlap or nearly overlap when the p-value is less than 0.05 whereas, on the contrary do not overlap when p=0.1. Please check.

Is the study design appropriate and does the work have academic merit?

Partly

Is the work clearly and accurately presented and does it cite the current literature?

Partly

If applicable, is the statistical analysis and its interpretation appropriate?

Partly

Are all the source data underlying the results available to ensure full reproducibility?

Yes

Are the conclusions drawn adequately supported by the results?

Partly

Are sufficient details of methods and analysis provided to allow replication by others?

Partly

Reviewer Expertise:

Sensory perception, eating behaviour. I have some knowledge in statistics and I made a number of comments on the statistical analyses. However, the comments from a specialist could be useful.

I confirm that I have read this submission and believe that I have an appropriate level of expertise to confirm that it is of an acceptable scientific standard.

# Reviewer report: Reviewer response for version 2

**DOI:** 10.21956/openreseurope.16029.r29894

**Published:** 2022-08-30

**Authors:** Melania Melis

**Affiliations:** 1 Department of Biomedical Sciences, University of Gastronomic Sciences, Caligari, Italy

Authors accurately address all the comments according to my suggestions and the manuscript has been significantly improved. The manuscript is now suitable for indexing.

Is the study design appropriate and does the work have academic merit?

Yes

Is the work clearly and accurately presented and does it cite the current literature?

Yes

If applicable, is the statistical analysis and its interpretation appropriate?

Yes

Are all the source data underlying the results available to ensure full reproducibility?

Yes

Are the conclusions drawn adequately supported by the results?

Yes

Are sufficient details of methods and analysis provided to allow replication by others?

Partly

Reviewer Expertise:

NA

I confirm that I have read this submission and believe that I have an appropriate level of expertise to confirm that it is of an acceptable scientific standard.

# Response

**Published:** 2022-09-27

**Authors:** Ervina Ervina

**Affiliations:** Food Technology Department, Faculty of Engineering, Bina Nusantara University, Jakarta, Indonesia 11480, Norway

Dear Reviewer, 

 Thank you for the feedback we are grateful to receive your comments and feedback for the manuscript improvement.

# Reviewer report: Reviewer response for version 2

**DOI:** 10.21956/openreseurope.16029.r29824

**Published:** 2022-08-22

**Authors:** Sylvie Issanchou

**Affiliations:** 1 Centre des Sciences du Goût et de l'Alimentation, CNRS, INRAE, Institut Agro, Université de Bourgogne, F-21000 Dijon, France

I thank the authors for their revision. The introduction has been improved, the objectives are now much clearer. However, I have still some comments, in particular on the presentation of the analyses and of the results. In fact, it is not obvious to find the results of all analyses mentioned in the data analysis section. The discussion must also be revised. In fact, some explanations do not fit with the absence of significant association between taste sensitivities and food propensity.

**Introduction**

- The first two paragraphs cover a same topic, i.e., the association between children’s taste sensitivity and their eating behavior. Thus, they should be merged. This would avoid some redundancies.
- End of the second paragraph: “In children aged 12–13 years a more frequent consumption of fast food was associated with decreasing sensitivity to saltiness and, as a consequence, their preference for saltier beansprout soups increased (Kim & Lee, 2009)”. In this sentence using the verbs 'decrease' and 'increase' may suggest that Kim & Lee’s study is a longitudinal study. It would be better to mention a ‘lower sensitivity’ and ‘a higher preference’.
- In the third paragraph, you mix different points. Firstly, you refer to exposure during early childhood but you studied preadolescents and did not evaluate their early exposure. Thus, this part should be deleted. Secondly, you explain why you had a specific focus on dairy products but this point is also mentioned at the end of the introduction. Thus, it would be more logical to move the sentences referring to the consumption of dairy products at the end of the introduction. Moreover, it would be more appropriate to speak about frequency of consumption of dairy foods rather than exposure.

**Methods**

- Page 4, end of the first column “preadolescents have different intensity and liking perceptions for these two compounds (Ervina *et al*., 2020)”. Since, here you measure detection thresholds it would be more logical to refer to your previous result on the moderate correlations between the detection thresholds of these two compounds (“The sensitivities to bitterness from caffeine and quinine had significant moderate correlations for … DT (r = 0.38, p < 0.001)) than to intensity and liking perceptions.
- Since you have merged the underweight (one preadolescent) and normal weight individuals, you should name the group UN/NO and not only NO. More importantly, you should check if excluding the underweighted girl would modify your results. Moreover, in Table 1, you should add, the mean, min and max of the z-BMI for each group.
- Power analysis: Since, you performed many analyses, one can wonder if your conclusion about the power analysis is true for all the analyses.

**Data analysis and Results**

- PCA: The PCA is not useful and what is especially troublesome is that it leads you to interpretations that contradict other analyses which are more relevant (page 7: “The PCA biplot (Figure 2) also displays positive associations between BMI and detection thresholds, where the detection thresholds were included as supplementary variables.” Then, “Based on linear regression, our results did not show a significant influence of taste sensitivity on children’s BMI.”). This is certainly related to the fact that the 1 ^st^ plan of this PCA explains only 53% of the variance. Moreover, examination of a PCA plot is not appropriate to conclude about the significant association. Thus, the conclusions of the Mann-Whitney are more relevant than those from the PCA. Consequently, this PCA must be deleted.
- Why did you include the BMI variable (z-BMI score, I suppose) only when you analyse the association between taste sensitivity and FPQ of dairy products and not in the other ANOVAs?
- Page 6, top of the column: the sentences “The association between FPQ of dairy products and taste sensitivity was also evaluated using mixed model ANOVA. … The significant differences across different BMI groups for each dairy food item were further computed using a Mann-Whitney test.” were duplicated. My comment on your first version was not clear. In fact, I mean that if the conditions of application of parametric tests are not respected, non-parametric tests could be more appropriate. However, my comment was not only for group comparison but also (and mainly) for using ANOVA. If your data fit the condition for a parametric analysis (ANOVA), it is not logical to compare groups with a non-parametric analysis.
- Page 6, the sentences “The association between taste sensitivity and BMI was investigated using linear regression models. The models were … variables, BMI score was employed as a response variable” should be moved at the end of the 1 ^st^ paragraph on data analysis since this analysis does not concern the Food Propensity Questionnaire.

**Results**

- General comment: you should present, at least in supplementary tables, the details of the results of your ANOVAs and regressions.
- Cronbach’s alpha of CEBQ: It is interesting to mention a good internal consistency for both food approach and food avoidance but these two dimensions are not defined and not further used (only mentioned in the discussion). As mentioned in my review of your first version, I wonder if it could be interesting to calculate a score for food approach (and food avoidance) and use these variables in your instead of all sub-dimensions.
- I may have missed something but I have not seen the results of the mixed model ANOVA with DFE as the response variable with the detection threshold level and the taste qualities (sweet, sour, salty, bitter-caffeine, and umami) as explanatory variables. Do you refer to this analysis when you write “Neither could we detect any significant effect of children’s basic taste sensitivity on FPQ score in general, showing that taste sensitivity threshold did not relate to frequency consumption of certain foods”? Please clarify.
- Again, I may have missed something but I have not seen the results of the five linear regression models computed with taste detection threshold as explanatory variable and FPQ scores (each basic taste) as response variables conducted to further investigate the effect of taste sensitivity on FPQ per taste (i.e., sweet foods, sour foods, salty foods, etc.).
- “However, when the BMI variable was involved in the model, there were significant differences in the FPQ for the selected dairy products between the normal weight and overweight/obese children.” There are several problems here. Firstly, here, not only you include the BMI group in the model but you consider the dairy products. So, this is quite different from the previous results. Moreover, the way you present the results does not seem to correspond to the models presented on the top of the second column on page 6. You did not mention any result about the association between FPQ of dairy products and taste sensitivity while this is the objective that you mention in the introduction (“Therefore, it is of interest to investigate the influence of children’s taste sensitivity on their exposure to dairy foods”).
- Figure 3: The last part of the legend of figure 3 (“*p≤0.1, **p≤0.05 based on mean value on Mann Whitney) is not at all appropriate since the Mann Whitney test is based on ranks, and not on means. However, as I mentioned above, if your data fit the condition for a parametric analysis, it is not logical to compare groups with a non-parametric analysis. On this figure you must indicate the meaning of the error bars (standard deviations?, standard errors?, confidence interval?). Moreover, based on these errors bars, it is surprising that for semi-hard cheese and low fat milk the p-values are ≤ 0.1 compared to other other foods for which p ≤ 0.05. This could be due to the fact the p-values correspond to the Mann-Whitney test but, please, check.

**Discussion**

- Associations between children’s basic taste sensitivity and eating behaviour: There are redundancies in the two first paragraphs (e.g., “bitter taste is “naturally” associated with poisonous substances and this taste helps humans to avoid ingestion of harmful foods). Moreover, the sentence “Bitter taste is commonly associated with food aversion” could be deleted since here you refer to food responsiveness and not to food fussiness. In fact, if you mention ‘food aversion’ the reader could expect an association between bitterness sensitivity and food fussiness. To avoid the redundancies, I wonder if it would not be simpler to present in one paragraph all associations with the sensitivity to bitterness. Here, you could refer to food approach.
  You write that “In addition, Ashi *et al.* (2017) reported that children aged 13–15 years who were less sensitive to sweetness had a significantly higher intake for sweet foods. These findings support the association between sweet taste sensitivity and emotional overeating behaviour found in our study”. I do not completely agree because you did not find any association with frequency of consumption of sweet foods. This point must be discussed.
- Caffeine and quinine sensitivities and eating behavior: “Caffeine and quinine may not activate the same bitterness receptors, and this could result in differences in bitterness perception”: you could be more certain. In fact, according table 1 of the paper by Meyerhof et al., quinine has a different response profile than caffeine. However, quinine and caffeine stimulates the same set of hTAS2Rs but quinine stimulates 4 hTAS2Rs not stimulated by caffeine. So, your result is surprising.
  I agree that caffeine and quinine are not found in the same foods. However, since here you look at associations with dimensions of eating behaviour and not associations with consumption of specific foods, this comment is not really appropriate here.
- Relationship between basic taste sensitivity and FPQ: Your discussion is interesting and relevant but the flow is not perfectly logical. For example, the sentence “Moreover, preadolescents have been characterized as curious and autonomous eaters, but their drinking and eating habits are still framed by their parental food practices (Nicklaus, 2020)” should be rephrased. You could delete the last part of this sentence (redundant with the previous one) and write, for example, “However, preadolescents have been characterized as curious and autonomous eaters”. Then, you could include the results from USA and Norway and then include the sentence “The children’s eating frequency recorded by FPQ may not, however, capture what is children’s “actual” consumption but rather indicate what is “served” to them by their parents” after “This indicates that children may have eating activities outside home”. Finally, delete “and these might not be recorded by the questionnaire.”
- Preadolescents’ BMI: The fact that your results showed a higher number of overweight (28%) compared to the literature and the fact that in this study almost 80% of the parents in this study had a high education level (bachelor’s degree or higher) is quite contradictory with the fact that the risk of overweight is higher when parents had a lower education. This point must be underlined.
- Association between dairy food propensity and children’s BMI: I wonder if the Norwegian Directorate of Health (2016) recommends low-fat milk for regular consumption whatever the age because below 2 years of age, fat consumption is recommended.
  You indicate the significant differences for fermented milk, skimmed milk, and flavoured milk and then you report differences between BMI groups for low-fat milk and semi-hard cheese. I understand that you make a difference because the p-values are <0.05 for fermented milk, skimmed milk, and flavoured milk and only <0.1 for the two other dairy foods. However, this is strange. If p>0.05, you should not comment results, but one can accept that you comment results with p<0.10 but clearly as tendencies.
  I agree with your explanations concerning the differences between BMI groups concerning low fat milk, flavoured milk and semi-hard cheese, but the direction of difference concerning skimmed milk is surprising.
  You could not simply say that the results of the recent systematic and meta-analysis review by Vanderhout *et al*. (2020) suggesting that higher consumption of whole milk is associated with low adiposity in childhood could be related to the results found in our study. The problem is that when reading your sentence one can conclude that higher consumption of whole milk leads to low adiposity in childhood, a hypothesis discussed by Vanderhout et al. But, this is not what you conclude. Thus, if you quote this paper, you must expand your explanations and indicate that Vanderhout et al mentioned possible reverse causality and the fact that parents of children who have higher adiposity might choose lower-fat milk to reduce the risk of overweight or obesity as you did.
  Relationships between preadolescents’ basic taste sensitivity, BMI, and eating behaviour: As mentioned previously delete the PCA and consequently all the discussion based on PCA.
  “This indicates that taste sensitivity could mediate the complex interplay between eating behaviour and BMI in preadolescent children.”: I do not understand this hypothesis on the mediation role of taste sensitivity. In fact, you found an association between taste sensitivity and eating behaviour assessed by the CEBQ. You also found, as previous authors, the association between some eating behaviour dimensions and BMI. However, you did not find an association between taste sensitivity and BMI. This would mean that taste sensitivity would not directly influence BMI but would impact BMI through eating behaviour.
- Study limitations: Thank for the modifications made on this section.

**Minor points and typos**

- Abstract: The last sentence of the conclusions is incomplete.
- When you refer to BMI or BMI score, indicate clearly if you refer to the z-BMI score.
- Legend of figure: It seems that the last sentence (“Relationships between eating behaviour (CEBQ), food propensity (FPQ), and weight status (BMI)”) does not refer to this figure.
- In my previous review, I made a comment on the term “propensity”. You answered that you have changed the term of propensity in the manuscript and that to prevent misinterpretation, you have used “food frequency” in the keywords. But I have not seen this change in the keywords and/or in the text.

Is the study design appropriate and does the work have academic merit?

Partly

Is the work clearly and accurately presented and does it cite the current literature?

Partly

If applicable, is the statistical analysis and its interpretation appropriate?

Partly

Are all the source data underlying the results available to ensure full reproducibility?

Yes

Are the conclusions drawn adequately supported by the results?

Partly

Are sufficient details of methods and analysis provided to allow replication by others?

Partly

Reviewer Expertise:

Sensory perception, eating behaviour. I have some knowledge in statistics and I made a number of comments on the statistical analyses. However, the comments from a specialist could be useful.

I confirm that I have read this submission and believe that I have an appropriate level of expertise to confirm that it is of an acceptable scientific standard, however I have significant reservations, as outlined above.

# Response

**Published:** 2024-01-12

**Authors:** Ervina Ervina

**Affiliations:** Food Technology Department, Faculty of Engineering, Bina Nusantara University, Jakarta, Indonesia 11480, Norway

**We thank the reviewer for the effort and time in this second review process. We adjusted our third version of the manuscript at our best following the advice. We believe the manuscript has improved in clarity and consistency. We also would like to say sorry that it takes long time to finalized this 3rd version. **   Reviewer: Sylvie Issanchou **I thank the authors for their revision. The introduction has been improved, the objectives are now much clearer. However, I have still some comments, in particular on the presentation of the analyses and of the results. In fact, it is not obvious to find the results of all analyses mentioned in the data analysis section. The discussion must also be revised. In fact, some explanations do not fit with the absence of significant association between taste sensitivities and food propensity.** We thank the reviewer for the effort and time in this second review process. We adjusted our third version of the manuscript at our best following the advice. We believe the manuscript has improved in clarity and consistency.   INTRODUCTION **The first two paragraphs cover a same topic, i.e., the association between children’s taste sensitivity and their eating behavior. Thus, they should be merged. This would avoid some redundancies.** We have merged these two paragraphs together and have deleted redundant parts. **End of the second paragraph: “In children aged 12–13 years a more frequent consumption of fast food was associated with decreasing sensitivity to saltiness and, as a consequence, their preference for saltier beansprout soups increased (Kim & Lee, 2009)”. In this sentence using the verbs 'decrease' and 'increase' may suggest that Kim & Lee’s study is a longitudinal study. It would be better to mention a ‘lower sensitivity’ and ‘a higher preference’.** The study by Kim & Lee (2009) is not a longitudinal study. We have changed the term of “increase” and “decrease” as suggested. The sentences now have been adjusted into: “In children aged 12–13 years a more frequent consumption of fast food was associated with lower sensitivity to saltiness and, as a consequence, the subjects showed a higher preference for saltier beansprout soups” **In the third paragraph, you mix different points. Firstly, you refer to exposure during early childhood but you studied preadolescents and did not evaluate their early exposure. Thus, this part should be deleted. Secondly, you explain why you had a specific focus on dairy products but this point is also mentioned at the end of the introduction. Thus, it would be more logical to move the sentences referring to the consumption of dairy products at the end of the introduction. Moreover, it would be more appropriate to speak about frequency of consumption of dairy foods rather than exposure** The sentence regarding exposure in early childhood has been deleted. In addition, the introduction regarding the frequency of consumption of dairy food in this part has been moved to the end of the paragraph. We have changed the term “exposure” into “frequency of consumption”. **METHODS** **Page 4, end of the first column “preadolescents have different intensity and liking perceptions for these two compounds (Ervina et al., 2020)”. Since, here you measure detection thresholds it would be more logical to refer to your previous result on the moderate correlations between the detection thresholds of these two compounds (“The sensitivities to bitterness from caffeine and quinine had significant moderate correlations for … DT (r = 0.38, p < 0.001)) than to intensity and liking perceptions** We have revised the sentence by highlighting the correlation between detection and recognition thresholds of the bitter compounds based on our previous study: “Both the bitter compounds of caffeine and quinine were considered in this study, because our previous results suggested that preadolescents have a moderate correlation between these two bitter compounds for the detection and recognition threshold levels. **Since you have merged the underweight (one preadolescent) and normal weight individuals, you should name the group UN/NO and not only NO. More importantly, you should check if excluding the underweighted girl would modify your results. Moreover, in Table 1, you should add, the mean, min and max of the z-BMI for each group.** We have now merged underweight (UN) and normal weight (NO) children, however, only one child was reported as underweight. The result is similar with or without the UN subject, therefore we have included the UN subject in the analysis. The mean, min, and max of the z-BMI have been added in table 1. **Power analysis: Since, you performed many analyses, one can wonder if your conclusion about the power analysis is true for all the analyses.** The power analysis was conducted for the ANOVA and linear regression model in general with a medium effect size. This information has been added in the manuscript **DATA ANALYSIS and RESULTS** **PCA: The PCA is not useful and what is especially troublesome is that it leads you to interpretations that contradict other analyses which are more relevant (page 7: “The PCA biplot (Figure 2) also displays positive associations between BMI and detection thresholds, where the detection thresholds were included as supplementary variables.” Then, “Based on linear regression, our results did not show a significant influence of taste sensitivity on children’s BMI.”). This is certainly related to the fact that the 1st plan of this PCA explains only 53% of the variance. Moreover, examination of a PCA plot is not appropriate to conclude about the significant association. Thus, the conclusions of the Mann-Whitney are more relevant than those from the PCA. Consequently, this PCA must be deleted** We have now deleted the PCA results and their discussion to avoid contradictory results as suggested by the reviewer. This also answers  comment no. 25 **Why did you include the BMI variable (z-BMI score, I suppose) only when you analyse the association between taste sensitivity and FPQ of dairy products and not in the other ANOVAs** As we had mentioned in our previous comment – since the group between UN/NO and OV/OB did not have a balanced number, we involved this parameter in the evaluation of the consumption frequency of dairy foods only. **Page 6, top of the column: the sentences “The association between FPQ of dairy products and taste sensitivity was also evaluated using mixed model ANOVA. … The significant differences across different BMI groups for each dairy food item were further computed using a Mann-Whitney test.” were duplicated. My comment on your first version was not clear. In fact, I mean that if the conditions of application of parametric tests are not respected, non-parametric tests could be more appropriate. However, my comment was not only for group comparison but also (and mainly) for using ANOVA. If your data fit the condition for a parametric analysis (ANOVA), it is not logical to compare groups with a non-parametric analysis** The double sentence has been deleted.  The student t-test was applied to compare groups. We believe that our data fit with a condition for the parametric test. We have checked the normality test, residual distribution, and data variance in the analysis **Page 6, the sentences “The association between taste sensitivity and BMI was investigated using linear regression models. The models were … variables, BMI score was employed as a response variable” should be moved at the end of the 1st paragraph on data analysis since this analysis does not concern the Food Propensity Questionnaire.** This now has been moved to the first paragraph of the data analysis section. **RESULTS** **General comment: you should present, at least in supplementary tables, the details of the results of your ANOVAs and regressions** We have added this summary as Table 2 which can be seen now in the results section **Cronbach’s alpha of CEBQ: It is interesting to mention a good internal consistency for both food approach and food avoidance but these two dimensions are not defined and not further used (only mentioned in the discussion). As mentioned in my review of your first version, I wonder if it could be interesting to calculate a score for food approach (and food avoidance) and use these variables in your instead of all sub-dimensions** We have considered incorporating all aspects based on the original CEBQ since the questionnaire has been validated. We have also conducted linear regression for each dimension of CEBQ in relation to taste sensitivity per taste and not based on these two dimensions. **I may have missed something but I have not seen the results of the mixed model ANOVA with DFE as the response variable with the detection threshold level and the taste qualities (sweet, sour, salty, bitter-caffeine, and umami) as explanatory variables. Do you refer to this analysis when you write “Neither could we detect any significant effect of children’s basic taste sensitivity on FPQ score in general, showing that taste sensitivity threshold did not relate to frequency consumption of certain foods”? Please clarify** Yes, the results from the mixed model ANOVA to investigate the general relationships between taste sensitivity and consumption frequency did not show any significant effect. The complete result is now summarized in Table 2 **Again, I may have missed something but I have not seen the results of the five linear regression models computed with taste detection threshold as explanatory variable and FPQ scores (each basic taste) as response variables conducted to further investigate the effect of taste sensitivity on FPQ per taste (i.e., sweet foods, sour foods, salty foods, etc.)** The results did not show any significant effects of each basic taste sensitivity on the consumption frequency of certain foods. We have now added some text in the manuscript: “Moreover, we could not detect any significant effect of children’s basic taste sensitivity on FPQ score in general, showing that taste sensitivity threshold did not relate to consumption frequency of corresponding tastes in foods (sweet foods, salty foods, sour foods, etc.)”. The results are now summarized in table 2 **“However, when the BMI variable was involved in the model, there were significant differences in the FPQ for the selected dairy products between the normal weight and overweight/obese children.” There are several problems here. Firstly, here, not only you include the BMI group in the model but you consider the dairy products. So, this is quite different from the previous results. Moreover, the way you present the results does not seem to correspond to the models presented on the top of the second column on page 6. You did not mention any result about the association between FPQ of dairy products and taste sensitivity while this is the objective that you mention in the introduction (“Therefore, it is of interest to investigate the influence of children’s taste sensitivity on their exposure to dairy foods”).**

   We have now moved these explanations to the second paragraph to separate the results.  The results of taste sensitivity and frequency consumption of dairy foods have been added to the text. “The results also showed no significant effect of taste sensitivity on consumption frequency of dairy foods. However, when the BMI variable was involved in the model,…” Figure 3: The last part of the legend of figure 3 (“*p≤0.1, **p≤0.05 based on mean value on Mann Whitney) is not at all appropriate since the Mann Whitney test is based on ranks, and not on means. However, as I mentioned above, if your data fit the condition for a parametric analysis, it is not logical to compare groups with a non-parametric analysis. On this figure you must indicate the meaning of the error bars (standard deviations?, standard errors?, confidence interval?). Moreover, based on these errors bars, it is surprising that for semi-hard cheese and low fat milk the p-values are ≤ 0.1 compared to other other foods for which p ≤ 0.05. This could be due to the fact the p-values correspond to the Mann-Whitney test but, please, check We have used student t-test now following our first version of the manuscript. The error bar stands for standard deviation and this information has been added in the footnote of figure 2. In fact, the results between Mann Whitney and t-test were quite similar **DISCUSSION** **Associations between children’s basic taste sensitivity and eating behaviour: There are redundancies in the two first paragraphs (e.g., “bitter taste is “naturally” associated with poisonous substances and this taste helps humans to avoid ingestion of harmful foods). Moreover, the sentence “Bitter taste is commonly associated with food aversion” could be deleted since here you refer to food responsiveness and not to food fussiness. In fact, if you mention ‘food aversion’ the reader could expect an association between bitterness sensitivity and food fussiness. To avoid the redundancies, I wonder if it would not be simpler to present in one paragraph all associations with the sensitivity to bitterness. Here, you could refer to food approach.** We have deleted the redundant part, especially the sentence of “Bitter taste is commonly associated with food aversion” We have deleted the “aversion” term following reviewer suggestion **You write that “In addition, Ashi et al. (2017) reported that children aged 13–15 years who were less sensitive to sweetness had a significantly higher intake for sweet foods. These findings support the association between sweet taste sensitivity and emotional overeating behaviour found in our study”. I do not completely agree because you did not find any association with frequency of consumption of sweet foods. This point must be discussed** In this part, we are aiming to explain the associations between children’s basic taste sensitivity and eating behaviour while the discussion related to consumption frequency was presented further forward. However, to not mix the discussion and confuse the reader we have deleted the references from Ashi et al. ***Caffeine and quinine sensitivities and eating behavior: “Caffeine and quinine may not activate the same bitterness receptors, and this could result in differences in bitterness perception”: you could be more certain. In fact, according table 1 of the paper by Meyerhof et al., quinine has a different response profile than caffeine. However, quinine and caffeine stimulates the same set of hTAS2Rs but quinine stimulates 4 hTAS2Rs not stimulated by caffeine. So, your result is surprising*** ***I agree that caffeine and quinine are not found in the same foods. However, since here you look at associations with dimensions of eating behaviour and not associations with consumption of specific foods, this comment is not really appropriate here.*** The paper of Meyerhof et al. is showcasing that from a total of 58 natural bitter compounds, they have different stimulation of the allele in hTAS2R. This suggests that each bitterness compound (including quinine and caffeine) triggered different alleles in molecular reception, indicating different bitterness profiles. Other references (Kamerud & Delwiche, 2007 and Briand & Salles, 2016) also support this statement. Moreover, we are using the study by Meyerhof et al. to refer that bitter taste has the largest number of compounds and receptors (responsible for bitter taste) compared to other basic tastes **Relationship between basic taste sensitivity and FPQ: Your discussion is interesting and relevant but the flow is not perfectly logical. For example, the sentence “Moreover, preadolescents have been characterized as curious and autonomous eaters, but their drinking and eating habits are still framed by their parental food practices (Nicklaus, 2020)” should be rephrased. You could delete the last part of this sentence (redundant with the previous one) and write, for example, “However, preadolescents have been characterized as curious and autonomous eaters”. Then, you could include the results from USA and Norway and then include the sentence “The children’s eating frequency recorded by FPQ may not, however, capture what is children’s “actual” consumption but rather indicate what is “served” to them by their parents” after “This indicates that children may have eating activities outside home”. Finally, delete “and these might not be recorded by the questionnaire.”** These sentences in this paragraph have been rephrased and adjusted following the reviewer’s suggestion. **Preadolescents’ BMI: The fact that your results showed a higher number of overweight (28%) compared to the literature and the fact that in this study almost 80% of the parents in this study had a high education level (bachelor’s degree or higher) is quite contradictory with the fact that the risk of overweight is higher when parents had a lower education. This point must be underlined** To avoid contradictory results, we have adjusted the sentences in this part. The sentence now becomes: “Their research highlighted a higher prevalence of overweight and/or obesity in children aged 6–11 years (17%) than in younger children aged 2–5 years (12.7%).” ***Association between dairy food propensity and children’s BMI: I wonder if the Norwegian Directorate of Health (2016) recommends low-fat milk for regular consumption whatever the age because below 2 years of age, fat consumption is recommended.*** ***You indicate the significant differences for fermented milk, skimmed milk, and flavoured milk and then you report differences between BMI groups for low-fat milk and semi-hard cheese. I understand that you make a difference because the p-values are <0.05 for fermented milk, skimmed milk, and flavoured milk and only <0.1 for the two other dairy foods. However, this is strange. If p>0.05, you should not comment results, but one can accept that you comment results with p<0.10 but clearly as tendencies*** Since our focus is preadolescents, we did not have data for children below two years old. We agree with the reviewer. Our results also clearly show that there was a “tendency” for a higher frequency consumption of low-fat milk for OV/OB group and lower frequency consumption for semi-hard cheese. This trend has been stated in the results section. **You could not simply say that the results of the recent systematic and meta-analysis review by Vanderhout et al. (2020) suggesting that higher consumption of whole milk is associated with low adiposity in childhood could be related to the results found in our study. The problem is that when reading your sentence one can conclude that higher consumption of whole milk leads to low adiposity in childhood, a hypothesis discussed by Vanderhout et al. But, this is not what you conclude. Thus, if you quote this paper, you must expand your explanations and indicate that Vanderhout et al mentioned possible reverse causality and the fact that parents of children who have higher adiposity might choose lower-fat milk to reduce the risk of overweight or obesity as you did.** We thank the reviewer for the suggestion. We have now adjusted the sentences to further discuss the findings by Venderhout et al. and try to bridge the relevancy with our discussion. **Relationships between preadolescents’ basic taste sensitivity, BMI, and eating behaviour: As mentioned previously delete the PCA and consequently all the discussion based on PCA.** We have now deleted all text related to the PCA analysis in the methods, results and discussion . **“This indicates that taste sensitivity could mediate the complex interplay between eating behaviour and BMI in preadolescent children.”: I do not understand this hypothesis on the mediation role of taste sensitivity. In fact, you found an association between taste sensitivity and eating behaviour assessed by the CEBQ. You also found, as previous authors, the association between some eating behaviour dimensions and BMI. However, you did not find an association between taste sensitivity and BMI. This would mean that taste sensitivity would not directly influence BMI but would impact BMI through eating behaviour.** Since this sentence was related to the PCA result it is now deleted.  (comments no. 8 and 25 above). **Study limitations: Thank for the modifications made on this section** We added this section considering the reviewer feedback in the previous version of the manuscript **MINOR POINTS** **Abstract: The last sentence of the conclusions is incomplete.** It seems that the sentences have been cut by the system (it has a limitation of 300 words only). Now we have ensured that the abstract won’t exceed the word limit. The complete sentence should be “The results may be used to develop effective strategies to promote healthy eating practices by considering taste sensitivity in preadolescents” **When you refer to BMI or BMI score, indicate clearly if you refer to the z-BMI score.** This has been now corrected in the results and methods sections. **Legend of figure: It seems that the last sentence (“Relationships between eating behaviour (CEBQ), food propensity (FPQ), and weight status (BMI)”) does not refer to this figure.** We have deleted this sentence **In my previous review, I made a comment on the term “propensity”. You answered that you have changed the term of propensity in the manuscript and that to prevent misinterpretation, you have used “food frequency” in the keywords. But I have not seen this change in the keywords and/or in the text** We have now changed this term for the entire manuscript into “frequency” as well as in the title

# Reviewer report: Reviewer response for version 1

**DOI:** 10.21956/openreseurope.15220.r27882

**Published:** 2021-12-02

**Authors:** Melania Melis

**Affiliations:** 1 Department of Biomedical Sciences, University of Gastronomic Sciences, Caligari, Italy

The study described in the manuscript titled *Basic taste sensitivity, eating behaviour, and propensity of dairy foods of preadolescent children: How are they related?* is well designed and well written. However, there are some points that need to be carefully elucidated before publication. Specific recommendations and questions are detailed below.

**Title:**

- Since the authors investigate the relationships between taste sensitivity, eating behaviour, and children’s body mass index, I would suggest this information in the title.

**Abstract:**

- Please add more information about the number of subjects recruited and the range of age in the methods section.

**Meth** **ods:**

 Participants section:

- Please add the range of age. The number of participants is quite small, the authors should indicate this as a limitation. However, did the authors conduct the power statistic to determine the sample size?

Children’s basic taste sensitivity measurement:

- The authors must explain why they chose to determine the detection threshold instead of the recognition threshold. The detection threshold is defined as the solution at which the subjects clearly indicated it as different from water but did not necessarily recognize the type of stimulus. The recognition threshold is defined as the solution at which the subjects clearly identified the type of stimulus, that is, the taste.
- The authors stated: *“rinse their mouth with water and to eat crackers to clean their palate between tastings of each cup.” *Why did the authors choose this procedure? Eating the crackers could make a bias in the experimental procedure. In addition, please specify the time of interstimulus interval.

Data analysis:

- The authors stated that “The classification for the weight status into obesity, overweight, normal, and underweight groups followed the BMI/age chart standard for school-age children based on WHO (2007)”.  I would suggest including more detail about this classification, did the authors classify into groups based on percentiles of the charts or the z-scores? It is also known that the classification is different for girls and boys, did the authors consider this factor for classification? Please add.
- The authors stated that “gender distribution was quite balanced across the BMI groups.” I would suggest performing a chi-square to test any differences in BMI/gender groups.

FPQ:

- Since in Figure 3 the authors perform the analyses by using the FPQ score, the authors must include in the methods how the FPQ score was determined.

**Results:**

- The authors must include one table with the principal characteristic of the panel (total number of subjects, n of female and male, n of normal/underweight and overweight/obese, age and so on).
- The background of Figure 1 must be changed; light symbols are not clearly visible. In order to point out these symbols the background must be white, please modify.
- Please specify what the legend count means. Is it the number of subjects with the same values?
- The authors stated that Figure 3 showed “The food frequency consumption (FPQ) of dairy food items according to children’s BMI (NO=normal weight children; OV/OB=overweight/obese children). Please be more precise, this figure showed “The food frequency consumption (FPQ) of dairy food items according to subjects classified as NO and OV/OB".

**Discussion:**

- The authors start the discussion with the children’s BMI even if this is not the main object of the manuscript. I would suggest start describing the results from the main purpose of the manuscript.

Is the study design appropriate and does the work have academic merit?

Yes

Is the work clearly and accurately presented and does it cite the current literature?

Yes

If applicable, is the statistical analysis and its interpretation appropriate?

Yes

Are all the source data underlying the results available to ensure full reproducibility?

Yes

Are the conclusions drawn adequately supported by the results?

Yes

Are sufficient details of methods and analysis provided to allow replication by others?

Partly

Reviewer Expertise:

Chemosensory perception; Individual differences; PROP tasting; Taste and olfaction genes; Taste modulation; Electrophysiological recordings from human tongue; Taste and olfaction and health.

I confirm that I have read this submission and believe that I have an appropriate level of expertise to confirm that it is of an acceptable scientific standard, however I have significant reservations, as outlined above.

# Response

**Published:** 2022-05-24

**Authors:** Ervina Ervina

**Affiliations:** Food Technology Department, Faculty of Engineering, Bina Nusantara University, Jakarta, Indonesia 11480, Norway

We are grateful to the reviewers for the time in reviewing and advising changes in the manuscript for improvement. We have tried to address all the comments to our best ability according to the suggestions and we believe that the manuscript has been improved. We provide the full details at our best of the changes and our responses below.

**Reviewer 2:** **Melania Melis **(University of Gastronomic Sciences, Caligari, Italy)

*The study described in the manuscript titled Basic taste sensitivity, eating behaviour, and propensity of dairy foods of preadolescent children: How are they related? is well designed and well written. However, there are some points that need to be carefully elucidated before publication. Specific recommendations and questions are detailed below* We thank the reviewer for the feedback 2 Tittle: *Since the authors investigate the relationships between taste sensitivity, eating behaviour, and children’s body mass index, I would suggest this information in the title.*

**Response: **This comment is corroborating with the reviewer 1. We have adjusted the paper’s title and have included the children’s BMI.

***Abstract***

*Please add more information about the number of subjects recruited and the range of age in the methods section.*

**Response: **This comment is corroborating with the reviewer 1. This information has been added both in the abstract and in the method section. However, we had words limitation for abstract, some of the information was placed in the method section

***Material and method***

*Please add the range of age. The number of participants is quite small, the authors should indicate this as a limitation. However, did the authors conduct the power statistic to determine the sample size?*

**Response: **The age of the participants has been added into the manuscript: mean aged 10.9 ± 0.2 years. We have addressed the limited number of participants as one of our limitations in the discussion in particular for the obese group.   We have also conducted the statistical power to determine the sample size. This information has been added in the manuscript (Line 339-341) “Power analysis using the software developed by Faul, Erdfelder, Buchner, and Lang (2009), shows that with 69 subjects the experiment has good statistical power (0.8) with medium effect size.”

*Children’s basic taste sensitivity measurement:* *The authors must explain why they chose to determine the detection threshold instead of the recognition threshold. The detection threshold is defined as the solution at which the subjects clearly indicated it as different from water but did not necessarily recognize the type of stimulus. The recognition threshold is defined as the solution at which the subjects clearly identified the type of stimulus, that is, the taste.*

**Response: **The detection threshold was chosen because we would like to focus on children’s objective absolute threshold to measure their taste sensitivity (Reed & Knaapila, 2010). Moreover, detection threshold is also preferred over the recognition to prevent “guessing” of the participants over the taste stimuli when they were not able to recognize it even at the highest level (Gomez et al. 2004). This information has been inserted into the manuscript including the additional references: “The detection threshold was preferred because we wanted primarily to measure the preadolescents’ physiological ability to perceive taste differences, rather than their cognitive ability to put a name on specific tastes (Gomez, Cassís-Nosthas, Morales-de-León, & Bourges, 2004; Reed & Knaapila, 2010).” Line 155-159

*Children’s basic taste sensitivity measurement:* *The authors stated: “rinse their mouth with water and to eat crackers to clean their palate between tastings of each cup.” Why did the authors choose this procedure? Eating the crackers could make a bias in the experimental procedure. In addition, please specify the time of interstimulus interval.*

**Response:** This practice was applied to avoid the strong lingering taste that may occur after children tasting the highest concentration (the strongest intensity, level 5). We have adjusted this information in the manuscript: “There was a short “break” for around three minutes before the participants changed to the next taste series (change stimuli)” Line 211-212 7

***Data analysis***

*The authors stated that “The classification for the weight status into obesity, overweight, normal, and underweight groups followed the BMI/age chart standard for school-age children based on WHO (2007)”.  I would suggest including more detail about this classification, did the authors classify into groups based on percentiles of the charts or the z-scores? It is also known that the classification is different for girls and boys, did the authors consider this factor for classification? Please add.*

**Response: **This comment corroborates with the comment from the reviewer 1. The BMI classification used z-scores and considered gender. This information has been added in the manuscript: “The classification for the weight status into obesity, overweight, normal, and underweight groups followed the BMI/age chart based on z-score for school-age children, the chart is separated for boys and girls respectively (WHO, 2007).” Line 286-288

*The authors stated that “gender distribution was quite balanced across the BMI groups.” I would suggest performing a chi-square to test any differences in BMI/gender groups.*

**Response: **This has been added in the manuscript “The differences for gender and BMI status were evaluated using chi-square analysis” (Line 291) and has been mentioned further in the results section (Line 356): “The gender distribution was balanced across the BMI groups (p-value > 0.05 based on chi-square analysis).”

***FPQ***

*Since in Figure 3 the authors perform the analyses by using the FPQ score, the authors must include in the methods how the FPQ score was determined.*

**Response: **The score of FPQ score has been described in the manuscript under the methods section: “The parent’s responses regarding FPQ of a certain food were recorded in an eating frequency with options of “never/rarely”, “1-3 times per month”, “1-3 times per week”, “4-6 times per week”, “daily”, and “more than once a day”.(Line 279-282). This data was further converted into DFE and the conversion of FPQ score into DFE (Daily Frequency Equivalent) was further explained in the data analysis section (Line 310-315)

***Results***

*The authors must include one table with the principal characteristic of the panel (total number of subjects, n of female and male, n of normal/underweight and overweight/obese, age and so on).*

**Response: **This table (Table 1) has been added in the manuscript following the reviewer’s suggestion.

*Please specify what the legend count means. Is it the number of subjects with the same values?*

**Response: **The count means number of children. The more saturated color is indicating more children with the same score of DT and eating behaviour. This information has been added in the legend of the manuscript (Figure 1)

*The authors stated that Figure 3 showed “The food frequency consumption (FPQ) of dairy food items according to children’s BMI (NO=normal weight children; OV/OB=overweight/obese children). Please be more precise, this figure showed “The food frequency consumption (FPQ) of dairy food items according to subjects classified as NO and OV/OB".*

**Response: **We thank the reviewer for the correction. This information in Figure 3 has been adjusted accordingly.

**Discussion**

*The authors start the discussion with the children’s BMI even if this is not the main object of the manuscript. I would suggest start describing the results from the main purpose of the manuscript.*

**Response: **This comment is in line with the comment of reviewer 1. We have moved this section further forward after the discussion of taste sensitivity and eating behaviour, and FPQ. The discussion now starts with taste sensitivity and eating behaviour relationships – which is the main interest in the present paper.

# Reviewer report: Reviewer response for version 1

**DOI:** 10.21956/openreseurope.15220.r27880

**Published:** 2021-11-22

**Authors:** Sylvie Issanchou

**Affiliations:** 1 Centre des Sciences du Goût et de l'Alimentation, CNRS, INRAE, Institut Agro, Université de Bourgogne, F-21000 Dijon, France

The links between taste perception and eating behavior are worthy of investigation in particular in pre-adolescent since there is not much data on this age range. However, the paper presents a number of weaknesses.

 The first and main point is that the objectives are not very clear. In the title, BMI is not mentioned while the authors look at the links between sensitivity for the different tastes and between BMI and the different dimensions of eating behavior. In the title, only daily foods are mentioned while in the paper frequency of consumption was evaluated and calculated for all food categories, even if there was a focus on dairy products. The rationale for focusing on dairy products is not obvious: why is it particularly interesting to examine the links between taste sensitivity and frequency of consumption of dairy foods because these foods have a significant contribution to Norwegian children’s daily intake? It could be interesting to examine the links between taste sensitivity and frequency of consumption of foods i) for which there is a quite large inter-individual variation of consumption, and ii) for foods which are either too much consumed such as sweet foods or not enough consumed such as vegetables. So, I suggest the authors to clarify and simplify their objectives. One option would be to focus on the links between taste perception and eating behavior while taking into account the BMI even if the size of your sample and the proportion of obese preadolescents is quite limited as you mentioned in your discussion.

 The second point is that the description of the statistical analysis is not clear and I think that the methods that are used are not necessarily the most appropriate (see detailed comments below).

**Abstract:**

- The added value of the present work is not clear when reading the part on the background. Is the key point that in the present study you focus on pre-adolescents?
- In line with my main general comment, there are different results that are presented and do not correspond to the aim presented in the background sub-paragraph.
- The mean and/or age range of the children are not indicated in the abstract.
- “while those who were less sensitive to sweetness and caffeine bitterness”: One can understand that you refer to individuals less sensitive to these two tastes (same comment for the discussion). Moreover, “while” is not really appropriate.

**Introduction:**

- The introduction should be strongly revised.
- The first paragraph indicates that within a group of children or preadolescents there is some variability in sensitivity for different taste compounds. First, this is true for adults as well as for elderly individuals. Moreover, looking at the variability is not the focus of your study. The first paragraph should immediately introduce your main objective.
- The second paragraph refers to the links between food sensitivity and consumption of different foods categories but the studies that are reported are sweet foods and bitter foods. This does not appear to correspond to your main objective (the one presented in the abstract) and dairy products are not mentioned.
- The third paragraph mixes different points. Moreover, eating behavior should be defined. In the sentence “The individual differences in perceiving taste at a genetic level were reported to be associated with eating behaviour (Chamoun *et al*., 2018; Hughes & Frazier-Wood, 2016).” it is not clear to what “eating behavior” refers to. In fact, the two papers refer to very different dimensions and not the eating behavior dimensions considered in your present study. Moreover, since you did not look at genetic differences, it is not necessary to introduce such a point in your introduction because it generates false expectations in readers. This could be done in the discussion.
- The fourth paragraph also mixes different points, and also presents questions: effect of exposure to foods with a specific taste on the modification of sensitivity to this taste, or on the change in liking for these foods and/or the taste of these foods), effect on the sensitivity to 6-n-propylthiouracil and acceptance of different dairy foods. Moreover, sometimes exposure refers to the frequency of consumption, and papers that are reported have examined the links between sensitivity and frequency of consumption (as in the second paragraph); so, these papers present cross-sectional studies. Other papers report longitudinal studies. Finally, here you introduced the interest of focusing on dairy products but this is not very convincing since you did not indicate that some preadolescents have an insufficient consumption of dairy foods. Moreover, dairy foods are a very large category of foods presenting very different tastes.

**Methods:**

- You invited to the study all the 6th grade cohort from the two schools, i.e.118 children and finally you had data for 69 children and parents. This is a quite small sample but you present this point as a limitation. However, one can wonder how you have determined the size of the sample. Did you conduct a power analysis? If not this suggestion must be given in your discussion.
- You wrote that “The schools were selected because of their location (13 km away) from the research institute (Nofima, Ås), aiming to minimize the possibility of the involvement of the parents who worked in the institute and knew the project”: it is strange because 13km is not very far away.
- The rationale for using two different bitter compounds, and more specifically caffeine and quinine, must be given.
- One can wonder why you did not give the preadolescents any information regarding the symbols and why you used symbols with individuals in this age range. Moreover, even if the participants did not receive any information about the symbols, they could guess that each series corresponds to a given taste.
- You must clarify how you determine the threshold: it is not clear if it was the lowest concentration where participants differentiate the sample from water or the lowest concentration with no more answers ‘water’ at higher concentrations.
- I do not understand why you justify using EyeQuestion. I am also surprised to read that SurveyMonkey is free.
- The part on the translation of the CEBQ should be presented before presenting the scale.

FPQ:

- You should give the reference of the original questionnaire.
- You indicate the total number of items (81) but you should indicate the number of food items for each of the nine categories.
- If you maintain to present data on the consumption of dairy products, the justification of this focus must be presented in the introduction before the objectives and not here. Here you should explain in which way you focus on dairy products: it is not clear if it was at the level of the questionnaire with including a more large number of dairy products than in the original questionnaire, or at the level of data analysis (in such a case, this must be presented in the section ‘Data analysis’.
- Categorizing the food items according to the basic taste profiles is a very interesting idea for looking at the link with sensitivity to the different tastes. It is not clear how you perform this classification because 3 classes of foods (out of the 6 classes defined by Martin *et al.*) were not characterized by a dominant taste but by several tastes. Moreover, the food taste database was developed on French foods; thus some foods of your FPQ questionnaire could be missing in the French food database and even if the foods were present, a Norwegian food item could have a different taste profile than the corresponding French food item. Thus, you must explain more clearly how you categorize the food items and, in the limitations, you must indicate that some differences could exist between the taste profile of the Norwegian foods and the French foods.

Data analysis:

- BMI: you mention BMI/age chart standard, but you should indicate that these charts are also different for girls and boys. Moreover, in the analyses it is not always clear if you used only the classification into 2 groups (i.e., normal/underweight and overweight/obese) based on percentiles of the charts or the BMI. In fact, you should use z-BMI and not BMI.
- For the association between taste sensitivity and the score for the different domains of the CEBQ, it would be important to check if there were no influent points and more globally if the conditions for using linear regression are respected. To answer this question, you conducted 48 different tests; so, a correction for multiple tests would be appropriate.
- Relationship between taste sensitivity and food propensity: the description of the ANOVA is not clear at all. Why detection threshold and taste as explanatory variables and not each taste threshold as an explanatory variable (so 5 explanatory variables). Moreover, in this analysis which FPQ scores did you use as a dependent variable: FPQ for each of the 81 food items? For each of the 9 food categories? Please, clarify.
- “To further investigate the effect of FPQ per food taste (i.e., sweet foods, sour foods, salty foods, etc.), five linear regression models were computed with taste detection threshold as explanatory variable and FPQ scores per food as response variables.”: this part also needs clarification. Do you mean that each food item was classified in a given taste category? Or did you calculate a score for all sweet (sour, …) foods? In both cases, how do define a food item as sweet (sour, …)?
- Association between FPQ for dairy products and taste sensitivity: I do not understand why dairy food items are explanatory variables since the FPQ score is calculated over the dairy products group. Moreover, the dairy food products is quite heterogeneous. In fact, plain yogurt is mainly sour, flavoured yogurt is mainly sweet, cheeses are mainly salty and some cheeses are also bitter. So, I do not understand the rationale of your analysis. Finally, why did you include BMI (category?, BMI value? BMI z-score?) in this analysis and not in the other analyses, in particular in those related to FPQ scores?
- Association between taste sensitivity and using linear regression models: firstly, this point was not mentioned in your objectives. Secondly, here you should use the z-BMI score and since these scores took into account the gender, it would not be necessary to include gender as a control variable and in this case, you could calculate correlations; so without making any assumption on the causality.
- PCA: this must be presented in the paragraph related on the analyses of CEBQ scores. Again, here z-BMI score must be used (not BMI).
- Student t-tests for comparing the BMI groups on each eating behaviour domain and for the consumption of each dairy food item: as for the linear regressions, one can wonder if the conditions of application of parametric tests are respected; non-parametric tests could be more appropriate.

**Results:**

- “The gender distribution was quite balanced across the BMI groups.”: a chi-square test should be conducted.
- Results concerning the Cronbach’s alpha must be given before the part (or at the beginning) on children’s taste sensitivity and eating behavior.
- “The PCA biplot (Figure 2) also displays positive associations between BMI and detection thresholds, where the detection thresholds were included as supplementary variables.”: this does not seem to be the case for umami.
- “FPQ score in general”: I do not understand what is this score.

**Discussion:**

- Since BMI is not mentioned as a main objective, it is strange that the discussion starts by BMI. Moreover, the data on other countries should not be part of the discussion of your paper.
- On the contrary, you could comment the values obtained on the different domains of CEBQ compared to previous published data.
- It seems that the following sentence “Previous studies also report that food intake in children could be modulated by negative emotions (Hill et al., 2018; Macht, 2008; Michels et al., 2012).” Is redundant with the beginning of this paragraph.
- “Further, several studies have suggested that low sensitivity to bitterness could increase children’s food intake (Goldstein *et al.*, 2007; Keller & Adise, 2016; Tepper *et al.*, 2011).”: Here you could develop the proposed underlying mechanisms.
- “Our results also show that taste sensitivity for sourness and bitterness (both caffeine and quinine) were significantly associated with desire to drink”: please, indicate the direction of the association.
- “Preadolescent children demonstrated individual differences in the perception of different bitter compounds such as caffeine and quinine, as was reported in a previous paper using data from the same participants (Ervina *et al.*, 2020)”: do your present data confirm this previous result?
- “The different bitter taste compounds have different bitterness profiles, and they elicit various intensity perceptions of bitterness”: Please, be indicate what the authors mean by “bitterness profiles”. Moreover, I am not sure that referring to differences of intensity is relevant here since you chose concentrations allowing you to determine detection thresholds.
- “Caffeine and quinine may not activate the same bitterness receptors”: based on the data published by Meyerhof *et al.*, you could be more precise.
- “Moreover, these two bitter compounds are not found in the same foods”: please, add a reference. Moreover, this part does not refer anymore to the domains of CEBQ but more a possible links between bitterness sensitivity and the frequency of consumption of specific foods.
- “Eating behaviour is influenced by many factors…”: this part would be more appropriate in a conclusion than here.
- “According to our results, no significant associations were found between taste detection threshold and FPQ score for any of the five taste modalities”: again, it is not clear to which FPQ score you refer.
- “In the USA, as many as 35% of children in early adolescence have been reported to eat outside home, with a big contribution of fast food (Reicks et al., 2015)”: do you have such data concerning Norway?
- “Correlations were found between children’s BMI and their eating behaviour based on the PCA mapping”: I have understood that the PCA was used to illustrate the links but then you performed t-tests to compare the different groups of preadolescents according to their weight status.
- “The PCA mapping also demonstrated that a higher detection threshold (lower taste sensitivity), may relate to a higher BMI”: here, it seems that you refer to a general taste sensitivity which is a point not presented in the results.
- Study limitations: you must add that you have collected cross-sectional data and thus for some results, in particular those related to the consumption of some food items, you could not conclude if the difference of consumption induced overweight/obesity or if overweight/obesity induced a modification of the food offered to the preadolescents.

**Conclusion:**

- Here you refer to food approach: one can wonder if it could be interesting to calculate a score for food approach. Based on the PCA, it seems that the four domains related to food approach are quite well correlated but, before calculating a score for food approach, you should check that it is relevant.

**Other comments:**

- Even if the term “propensity” has been used by the authors who developed the questionnaire, this term could be misinterpreted, and thus it would be better to speak about the frequency of consumption.
- In all your text, it will be better to refer to preadolescents instead of children when you refer to your own study.
- You should say “included” instead of “involved” when you refer to a variable added in a model.
- Figure 1: the dots in the lightest grey are not well visible.
- Figure 3: here you also conducted multiple tests. So, you should not mention and comment p values above 0.05 and below or equal to 0.10.

Is the study design appropriate and does the work have academic merit?

Partly

Is the work clearly and accurately presented and does it cite the current literature?

Partly

If applicable, is the statistical analysis and its interpretation appropriate?

Partly

Are all the source data underlying the results available to ensure full reproducibility?

Yes

Are the conclusions drawn adequately supported by the results?

Partly

Are sufficient details of methods and analysis provided to allow replication by others?

Partly

Reviewer Expertise:

Sensory perception, eating behaviour. I have some knowledge in statistics and I made a number of comments on the statistical analyses. However, the comments from a specialist could be useful.

I confirm that I have read this submission and believe that I have an appropriate level of expertise to confirm that it is of an acceptable scientific standard, however I have significant reservations, as outlined above.

# Response

**Published:** 2022-05-24

**Authors:** Ervina Ervina

**Affiliations:** Food Technology Department, Faculty of Engineering, Bina Nusantara University, Jakarta, Indonesia 11480, Norway

We are grateful to the reviewers for the time in reviewing and advising changes in the manuscript for improvement. We have tried to address all the comments to our best ability according to the suggestions and we believe that the manuscript has been improved. We provide the full details at our best of the changes and our responses below.

**Reviewer 1: Sylvie Issanchou (**Centre des Sciences du Goût et de l'Alimentation, AgroSup Dijon, CNRS, INRAE, Université Bourgogne Franche-Comté, Dijon, France)

***1:*** *The links between taste perception and eating behavior are worthy of investigation in particular in pre-adolescent since there is not much data on this age range. However, the paper presents a number of weaknesses.* *The first and main point is that the objectives are not very clear. In the title, BMI is not mentioned while the authors look at the links between sensitivity for the different tastes and between BMI and the different dimensions of eating behavior. In the title, only daily foods are mentioned while in the paper frequency of consumption was evaluated and calculated for all food categories, even if there was a focus on dairy products. The rationale for focusing on dairy products is not obvious: why is it particularly interesting to examine the links between taste sensitivity and frequency of consumption of dairy foods because these foods have a significant contribution to Norwegian children’s daily intake? It could be interesting to examine the links between taste sensitivity and frequency of consumption of foods i) for which there is a quite large inter-individual variation of consumption, and ii) for foods which are either too much consumed such as sweet foods or not enough consumed such as vegetables. So, I suggest the authors to clarify and simplify their objectives. One option would be to focus on the links between taste perception and eating behavior while taking into account the BMI even if the size of your sample and the proportion of obese preadolescents is quite limited as you mentioned in your discussion.*

**Response: **We thank the reviewer for the feedback and suggestions. We have adjusted our objective and try to make it concise. The BMI is also mentioned in the title now. The title has changed to: “Basic taste sensitivity, eating behaviour, food propensity and BMI of preadolescent children: How are they related?” Dairy foods are emphasized because this food category is important and contributes as a major constituent in the Norwegian diet.

***2:*** *The second point is that the description of the statistical analysis is not clear and I think that the methods that are used are not necessarily the most appropriate (see detailed comments below).*

**Response: **The statistical analysis has been adjusted following the reviewer suggestion. We have used the multiple correction test for the adjustment of the analysis in particular for the association between taste sensitivity and eating behaviour. The data analysis section has been separated into subheadings for better explaining the statistical analysis for each variable of interest.

***Abstract*****

***3:*** *The added value of the present work is not clear when reading the part on the background. Is the key point that in the present study you focus on pre-adolescents?*

**Response: **We have modified the background in the manuscript in the abstract section. However, we could not insert more explanation due to words limitation “…., but to date there is limited study on preadolescents’ taste sensitivity and eating behaviour. This study aimed to investigate the associations….” (Line 22-23)  

***4:*** *In line with my main general comment, there are different results that are presented and do not correspond to the aim presented in the background sub-paragraph.*

**Response: **We have adjusted the sentence following the new tittle and have inserted the BMI and food propensity variables: “This study aimed to investigate the associations between taste sensitivity, eating behaviour, food propensity and BMI (Body Mass Index) in preadolescents.” Line 22-24

***5:*** *The mean and/or age range of the children are not indicated in the abstract*.

**Response: **The age range of the children has been inserted into the abstract: “A total of 69 child-parent dyads participated (preadolescents mean age =10.9 years).” (Line 30-31)

***6: “*** *while those who were less sensitive to sweetness and caffeine bitterness”: One can understand that you refer to individuals less sensitive to these two tastes (same comment for the discussion). Moreover, “while” is not really appropriate.*

**Response: **The word “while” has been removed from the text: “Those who were less sensitive to caffeine bitterness and to sweetness had higher emotional overeating scores” (Line 35-36)

**Introduction**

***7:*** *The introduction should be strongly revised*.

**Response: **We have changed and adjusted the introduction section following the reviewer’s suggestion

***8:*** *The first paragraph indicates that within a group of children or preadolescents there is some variability in sensitivity for different taste compounds. First, this is true for adults as well as for elderly individuals. Moreover, looking at the variability is not the focus of your study. The first paragraph should immediately introduce your main objective.*

**Response: **The first paragraph has been revised, we have merged paragraphs 1 and 2 and have adjusted the highlight focusing more into taste sensitivity, preference and BMI. The part on the variability in taste sensitivity has been partly removed from this section.

***9:*** *The second paragraph refers to the links between food sensitivity and consumption of different foods categories but the studies that are reported are sweet foods and bitter foods. This does not appear to correspond to your main objective (the one presented in the abstract) and dairy products are not mentioned*

**Response: **The second paragraph has been revised and merged with paragraph 1. In addition, the focus on dairy products has been inserted. New references have also been added: “Moreover, Maslin et al. (2016) report that children aged 11-12 years have different taste preferences according to their consumption of dairy foods. The study concludes that children with diets excluding dairy milk had a higher preference for bitter taste compared to children who consumed dairy milk. Dairy products constitute one of the main structures in Norwegian children's diet at age 9 to 13 years (Hansen, Myhre, Johansen, Paulsen, & Andersen, 2016). In addition, the dairy food category provides a diverse range of essential nutrients that are highly important for children’s growth and development (Givens, 2020). Therefore, it is of interest to investigate the influence of children’s taste sensitivity on their exposure to dairy foods. (Line 96-104)

***10:*** *The third paragraph mixes different points. Moreover, eating behavior should be defined. In the sentence “The individual differences in perceiving taste at a genetic level were reported to be associated with eating behaviour (Chamoun et al., 2018; Hughes & Frazier-Wood, 2016).” it is not clear to what “eating behavior” refers to. In fact, the two papers refer to very different dimensions and not the eating behavior dimensions considered in your present study. Moreover, since you did not look at genetic differences, it is not necessary to introduce such a point in your introduction because it generates false expectations in readers. This could be done in the discussion The term of eating behaviour in this paper has been narrowed into food choice and food consumption which may influence food preferences and liking following a review paper by DeCosta, Moller, Frost, & Olsen (2017).*

**Response: **This info has been inserted into the text (Line 77-78) We agree with the reviewer’s suggestion regarding the genetic differences. This info has been removed from the manuscript. We have also merged paragraphs 3 and 4.  

***11:*** *The fourth paragraph also mixes different points, and also presents questions: effect of exposure to foods with a specific taste on the modification of sensitivity to this taste, or on the change in liking for these foods and/or the taste of these foods), effect on the sensitivity to 6-n-propylthiouracil and acceptance of different dairy foods. Moreover, sometimes exposure refers to the frequency of consumption, and papers that are reported have examined the links between sensitivity and frequency of consumption (as in the second paragraph); so, these papers present cross-sectional studies. Other papers report longitudinal studies. Finally, here you introduced the interest of focusing on dairy products but this is not very convincing since you did not indicate that some preadolescents have an insufficient consumption of dairy foods. Moreover, dairy foods are a very large category of foods presenting very different tastes*

**Response: **The term of food exposure in this paragraph has been changed into frequency consumption. The focus on dairy products has been added, a new reference regarding the highlight of dairy foods has been included (Line 96-104)  

**Methods**

***12:*** *You invited to the study all the 6th grade cohort from the two schools, i.e.118 children and finally you had data for 69 children and parents. This is a quite small sample but you present this point as a limitation. However, one can wonder how you have determined the size of the sample. Did you conduct a power analysis? If not this suggestion must be given in your discussion*.

**Response: **We thank the reviewer for the feedback. We have conducted the power analysis in our study. The sample size is sufficient to have a good statistical power (0.8) for medium effect. This information has been added in the manuscript. Line 154-157 “Power analysis using the software developed by Faul et al. (2009), shows that with 69 subjects the experiment has good statistical power (0.8) with medium effect size.” (Line 338-340)

***13:*** *You wrote that “The schools were selected because of their location (13 km away) from the research institute (Nofima, Ås), aiming to minimize the possibility of the involvement of the parents who worked in the institute and knew the project”: it is strange because 13km is not very far away*

**Response:  **The schools were chosen because they were located at a different municipality from our research institute, Nofima. We have adjusted the sentence in the manuscript: “The schools were chosen from a neighbouring municipality of the research institute to minimise the chance of involving parents affiliated with the institute” (142-144)

***14:*** *The rationale for using two different bitter compounds, and more specifically caffeine and quinine, must be given.*

**Response: **This information has been added into the manuscript. We have also inserted new references: “Both the bitter compounds of caffeine and quinine were considered in this study, because our previous study suggested that preadolescents have different intensity and liking perceptions for these two compounds (Ervina et al., 2020). Moreover, bitter taste has the largest number of taste compounds and taste receptors compared to the other four basic tastes (Briand & Salles, 2016; Meyerhof et al., 2010).” (Line 162-166)

***15:*** *One can wonder why you did not give the preadolescents any information regarding the symbols and why you used symbols with individuals in this age range. Moreover, even if the participants did not receive any information about the symbols, they could guess that each series corresponds to a given taste The use of symbols aimed to reduce the risk of errors both at tasting level for the children and at preparation/serving level for the experimenter*.

**Response: **We have added this explanation with new reference has been inserted on the manuscript: “The use of the symbols was preferred to three-digit-random codes, aiming to simplify the test for the preadolescents and to ensure correct data collection. With such a large number of cups to handle (36 samples per subject in total), the preadolescents may have confused codes and/or the experimenter may easily have made mistakes when serving the samples for each participant, since the samples were served in a randomized-balanced order across subjects. Visually, it is also much simpler to make sure that each child received six clouds, six stars etc., than making sure each child received six “344” cups, six “763” cups etc, thus increasing the reliability of the protocol. Moreover, a study by Galler, Næs, L. Almli, and Varela (2020) suggests that the use of symbols are preferred to code the samples to avoid confusion in children aged 6-9 years.” (Line 177-186)

***16:*** *You must clarify how you determine the threshold: it is not clear if it was the lowest concentration where participants differentiate the sample from water or the lowest concentration with no more answers ‘water’ at higher concentrations*.

**Response:  **The detection threshold was determined based on the lowest level where subjects were able to distinguish the sample from water, either by declaring a taste (which could be correct or erroneous) or by choosing “I don’t know” (i.e. not knowing the taste, but perceiving that it is not plain water) (Line 199-203)

***17:*** *I do not understand why you justify using EyeQuestion. I am also surprised to read that SurveyMonkey is free*

**Response:  **We have deleted this information to avoid confusion.

***18:*** *The part on the translation of the CEBQ should be presented before presenting the scale*

**Response: **This part has been moved before the scale of CEBQ presented. (Line 244-246)

**FPQ**

***19:*** *You should give the reference of the original questionnaire.*

**Response: ** This has been addressed in the manuscript “The FPQ questionnaire was adapted from a previous Norwegian dietary survey developed by Totland et al. (2012).” (Line 257)

***20:*** *You indicate the total number of items (81) but you should indicate the number of food items for each of the nine categories.*

 This information has been added into the manuscript (Line 260-264). The questionnaire is available as open access and has been inserted as extended data in the manuscript. The link is available here: https://doi.org/10.5281/zenodo.5468625

***21:*** *If you maintain to present data on the consumption of dairy products, the justification of this focus must be presented in the introduction before the objectives and not here. Here you should explain in which way you focus on dairy products: it is not clear if it was at the level of the questionnaire with including a more large number of dairy products than in the original questionnaire, or at the level of data analysis (in such a case, this must be presented in the section ‘Data analysis’.*

**Response: **The justification of the dairy product has been presented in the introduction section. We have put the focus for FPQ with dairy in the analysis. This has been clarified in the data analysis section.

***22:*** *Categorizing the food items according to the basic taste profiles is a very interesting idea for looking at the link with sensitivity to the different tastes. It is not clear how you perform this classification because 3 classes of foods (out of the 6 classes defined by Martin et al.) were not characterized by a dominant taste but by several tastes. Moreover, the food taste database was developed on French foods; thus some foods of your FPQ questionnaire could be missing in the French food database and even if the foods were present, a Norwegian food item could have a different taste profile than the corresponding French food item. Thus, you must explain more clearly how you categorize the food items and, in the limitations, you must indicate that some differences could exist between the taste profile of the Norwegian foods and the French foods.*

**Response: **We have addressed the cultural challenge in the limitation section. “Our study may also suffer from a cross-cultural issue. The FPQ was developed according to Norwegian foods, but the classification of the food according to its taste (sweet, sour, salty, bitter, umami) in this study was conducted based on a French food database. Although all the foods listed in the FPQ are available in the database, taste profiles may be different between Norwegian and French foods” (Line 606-610) We have also added the explanation regarding the taste classification of the food items in the manuscript: “The food items were then further categorized according to their basic taste profiles into sweet (35 food items), sour (11), salty (25), bitter (10), and umami (13) foods. This classification was based on the food taste profile database which follows a study by Martin, Visalli, Lange, Schlich, and Issanchou (2014). The food taste database consists of nearly 600 food items evaluated by trained panellists based on a Spectrum ^TM^-like profiling approach for the five basic tastes and fattiness. Note that in our study, the foods were categorized into multiple taste categories according to the database (e.g., grapefruit was rated as both bitter (score=4.3) and sour (score=3.9) in the database, therefore this food was classified as both bitter and sour in our analyses).” (Line 270-278)

***Data analysis*****

***23:*** *BMI: you mention BMI/age chart standard, but you should indicate that these charts are also different for girls and boys. Moreover, in the analyses it is not always clear if you used only the classification into 2 groups (i.e., normal/underweight and overweight/obese) based on percentiles of the charts or the BMI. In fact, you should use z-BMI and not BMI.*

**Response: **This feedback was corroborating with a comment from the reviewer 2. We have used z-BMI to classify the BMI status of the children. The BMI is adjusted according to age and gender (boys and girls). This information has been added into the manuscript: “The classification for the weight status into obesity, overweight, normal, and underweight groups followed the BMI per age and gender charts based on z-scores  (WHO, 2007).” Line 286-288 

***24:*** *For the association between taste sensitivity and the score for the different domains of the CEBQ, it would be important to check if there were no influent points and more globally if the conditions for using linear regression are respected.  To answer this question, you conducted 48 different tests; so, a correction for multiple tests would be appropriate.*

**Response: **We have checked the residual distribution and variance for taste sensitivity and eating behaviour data. They are normally distributed (residual) and homogenous (variance), this ensures us to use llinear regression in our analysis. In addition, we have used Bonferroni multiple testing correction in the test. This info has been inserted into the manuscript “A Bonferroni test was carried out for multiple testing correction. Residual distribution and variances were checked in the data analysis” Line 301

***25:*** *Relationship between taste sensitivity and food propensity: the description of the ANOVA is not clear at all. Why detection threshold and taste as explanatory variables and not each taste threshold as an explanatory variable (so 5 explanatory variables). Moreover, in this analysis which FPQ scores did you use as a dependent variable: FPQ for each of the 81 food items? For each of the 9 food categories?*

**Response: **Please, clarify In this analysis, the FPQ was categorized per taste (FPQ sweet food, FPQ sour food, FPQ salty food, etc.) based on the classification of food items described under FPQ explanation in the methodology part (Line 270-278) Through this analysis we would like to investigate if there is any influence of taste sensitivity (detection threshold) on the frequency consumption of certain food taste category.

***26:*** *“To further investigate the effect of FPQ per food taste (i.e., sweet foods, sour foods, salty foods, etc.), five linear regression models were computed with taste detection threshold as explanatory variable and FPQ scores per food as response variables.”: this part also needs clarification. Do you mean that each food item was classified in a given taste category? Or did you calculate a score for all sweet (sour, …) foods? In both cases, how do define a food item as sweet (sour, …)?*

**Response: **The classification of the food items based on the tastes followed the food taste profile database from Martin *et al.* (2014) as has been explained in Line 270-278 in the manuscript. This comment corroborates with the comment No. 22 and we have provided similar answers

***27:*** *Association between FPQ for dairy products and taste sensitivity: I do not understand why dairy food items are explanatory variables since the FPQ score is calculated over the dairy products group. Moreover, the dairy food products is quite heterogeneous. In fact, plain yogurt is mainly sour, flavoured yogurt is mainly sweet, cheeses are mainly salty and some cheeses are also bitter. So, I do not understand the rationale of your analysis. Finally, why did you include BMI (category?, BMI value? BMI z-score?) in this analysis and not in the other analyses, in particular in those related to FPQ scores?*

**Response: **This analysis aimed to evaluate whether taste sensitivity (based on detection threshold) was able to influence the frequency consumption of dairy foods. We have also taken into account that dairy foods have different taste profiles, therefore the dairy food items were involved in the model. (Line 330-335) The BMI was based on BMI category (normal weight and overweight/obese). We have involved the BMI category variable in FPQ of dairy product aiming to highlight if there is any difference across BMI groups for the frequency consumption for this food group or not. However, we did not involve the BMI categories into other analysis of FPQ per taste (or to the specific food items) because we consider the unbalanced number of subjects between normal and overweight/obese groups in our study as our main limitation.

***28*** *: Association between taste sensitivity and using linear regression models: firstly, this point was not mentioned in your objectives. Secondly, here you should use the z-BMI score and since these scores took into account the gender, it would not be necessary to include gender as a control variable.*

**Response: **We have adjusted our objectives in the revised manuscript. The BMI has been clarified as z-BMI score. We have removed gender as control variable in the analysis.  

***29:*** *PCA: this must be presented in the paragraph related on the analyses of CEBQ scores. Again, here z-BMI score must be used (not BMI).*

**Response: **This has been moved in the section of CEBQ score analysis (line 303-307). In addition, the z-BMI score was also used in the analysis.

***30*** *: Student t-tests for comparing the BMI groups on each eating behaviour domain and for the consumption of each dairy food item: as for the linear regressions, one can wonder if the conditions of application of parametric tests are respected; non-parametric tests could be more appropriate.*

**Response: **We have changed the statistical analysis into a non-parametric Mann-Whitney test. The main findings remain the same.

**Results**

***31*** *: “The gender distribution was quite balanced across the BMI groups.”: a chi-square test should be conducted This comment corroborates with the comment from reviewer 2.*

**Response: **We have conducted the chi-square test in order to check the differences across gender. This information has been added into the manuscript: “The gender distribution was balanced across the BMI groups (p-value > 0.05 based on chi-square analysis).” (Line 356)

**32:** *Results concerning the Cronbach’s alpha must be given before the part (or at the beginning) on children’s taste sensitivity and eating behavior.*

**Response: **This information has been moved according to the reviewer’s suggestion (have been moved into line 360-363)

***33:** “FPQ score in general”: I do not understand what is this score.*

**Response: **This refers to the FPQ score for all of the tastes (FPQ sweet, salty, bitter, etc.). **The text in the manuscript has been adjusted. “Neither could we detect any significant effect of children’s basic taste sensitivity on FPQ score for all of tastes,…” Line 396

**Discussion**

***34:*** *Since BMI is not mentioned as a main objective, it is strange that the discussion starts by BMI. Moreover, the data on other countries should not be part of the discussion of your paper.*

**Response:** The discussion on children’s BMI has been moved accordingly. We also have deleted the discussion regarding the BMI comparison across countries in this section.

***35*** *: On the contrary, you could comment the values obtained on the different domains of CEBQ compared to previous published data The values regarding CEBQ data comparison with previous study has been presented in the relationships between children’s taste sensitivity, BMI and eating behaviour sections.*

**Response:**This is now explained in line 514-523.

***36:** It seems that the following sentence “Previous studies also report that food intake in children could be modulated by negative emotions (Hill et al., 2018; Macht, 2008; Michels et al., 2012).” Is redundant with the beginning of this paragraph.*

**Response:**To avoid redundancy, this sentence has been deleted.

***37*** *: “Further, several studies have suggested that low sensitivity to bitterness could increase children’s food intake (Goldstein et al., 2007; Keller & Adise, 2016; Tepper et al., 2011).”: Here you could develop the proposed underlying mechanisms.*

**Response: **We have added the proposed underlying mechanism. We have also included the new reference: “This may happen because bitter taste is “naturally” associated with poisonous substances and this taste helps humans to avoid ingestion of harmful foods (Mennella & Bobowski, 2015). By lowering the sensitivity of bitterness, the barrier to prevent the ingestion may also be disrupted and may increase food intake in general. This could explain the relationships found between sensitivity to bitterness and emotional overeating in this study.” (Line 444-449)

***38:*** *“Our results also show that taste sensitivity for sourness and bitterness (both caffeine and quinine) were significantly associated with desire to drink”: please, indicate the direction of the association.*

**Response: **The direction of the association has been inserted into the manuscript. ”… The preadolescents who were less sensitive to these tastes have higher scores for desire to drink.” Line *450-451*

***39*** *: “Preadolescent children demonstrated individual differences in the perception of different bitter compounds such as caffeine and quinine, as was reported in a previous paper using data from the same participants (Ervina et al., 2020)”: do your present data confirm this previous result?*

**Response:** We have presented this data in our previous published paper.  This info is presented in the manuscript: “Preadolescent children demonstrated individual differences in the perception of different bitter compounds such as caffeine and quinine, as was reported in our previous study using data from the same participants (Ervina et al., 2020).” Line 472-474

***40*** *: “The different bitter taste compounds have different bitterness profiles, and they elicit various intensity perceptions of bitterness”: Please, be indicate what the authors mean by “bitterness profiles”. Moreover, I am not sure that referring to differences of intensity is relevant here since you chose concentrations allowing you to determine detection thresholds. The profiles here may relate to the different (long) aftertaste of bitterness as well as the bitterness intensity.*

**Response: **We agree with the reviewer’s suggestion, since we use detection threshold and not intensity perception, the intensity may not be relevant here. However, this paragraph means to explain that the bitterness from different compounds can elicit different perception at the same concentration levels.

***41*** *: Caffeine and quinine may not activate the same bitterness receptors”: based on the data published by Meyerhof et al., you could be more precise.*

**Response: **We have added more information into the text. “Caffeine and quinine may not activate the same bitterness receptors, and this could result in variations in bitterness perception (Kamerud & Delwiche, 2007).” Line 479

***42*** *: “Moreover, these two bitter compounds are not found in the same foods”: please, add a reference.*

**Response: **The reference has been inserted into the text: “Moreover, these two bitter compounds are not found in the same foods (Briand & Salles, 2016)” (Line 481)

***43*** *: “Eating behaviour is influenced by many factors…”: this part would be more appropriate in a conclusion than here.*

**Response: **This part has been moved to the conclusion section (Line 631-637)

***44:*** *“According to our results, no significant associations were found between taste detection threshold and FPQ score for any of the five taste modalities”: again, it is not clear to which FPQ score you refer.*

**Response: **This FPQ score refers to each taste modality (FPQ for sweet foods, salty foods, sour foods, etc.). The information has been added in the manuscript. “…five taste modalities (i.e. sweet foods, salty foods,  bitter foods, etc.).” (Line 487)

***45:*** *“In the USA, as many as 35% of children in early adolescence have been reported to eat outside home, with a big contribution of fast food (Reicks et al., 2015)”: do you have such data concerning Norway?*

**Response: **We have added information regarding eating out in Norwegian school children in particular at schools. New references have been inserted as well: “In Norway, school lunch is typically brought from home, however, children aged 13 or more may buy food and drinks at the school or in its surroundings, and most of these food choices are considered to be unhealthy (Hilsen, Eikemo, & Bere, 2010). In addition, Norwegian school children aged 14-15 years buy soft drinks at school more than twice a week (Bere, Glomnes, Velde, & Klepp, 2007). This indicates that children may have eating activities outside home and these might not be recorded by the questionnaire.” Line 501-507

***46*** *“Correlations were found between children’s BMI and their eating behaviour based on the PCA mapping”: I have understood that the PCA was used to illustrate the links but then you performed t-tests to compare the different groups of preadolescents according to their weight status.*

**Response: **Indeed, the PCA was performed to investigate the link between variables and the student t-test has been changed into Mann-Whitney test and was analysed to compare the different groups according to the BMI status (normal vs. overweight/obese group)

***47:*** *“The PCA mapping also demonstrated that a higher detection threshold (lower taste sensitivity), may relate to a higher BMI”: here, it seems that you refer to a general taste sensitivity which is a point not presented in the results.*

**Response: **Yes, this refers to the general taste sensitivity which has been presented in Figure 2 in the results section. We have adjusted the sentence into: “The PCA mapping also demonstrated that in general, a higher detection threshold (lower taste sensitivity), may relate to a higher BMI, and this might be associated with the food-approach domain of CEBQ.” Line 583-585

***48:*** *Study limitations: you must add that you have collected cross-sectional data and thus for some results, in particular those related to the consumption of some food items, you could not conclude if the difference of consumption induced overweight/obesity or if overweight/obesity induced a modification of the food offered to the preadolescents.*

 We have added this limitation in the manuscript. A new reference has also been inserted. “In addition, this study is a cross-sectional study, therefore we are not able to conclude whether the different frequency consumption induced overweight or obesity or whether the overweight and obesity induced the frequency consumption. Moreover, the aetiology of overweight and obesity is multifaceted, complex and involves multiple factors (Lytle, 2009) thus assessing taste sensitivity solely may not be enough.” (Line 600-606)

**Other comments**

***49:*** *Even if the term “propensity” has been used by the authors who developed the questionnaire, this term could be misinterpreted, and thus it would be better to speak about the frequency of consumption.*

**Response: **We have changed the term of propensity in the manuscript. To prevent misinterpretation, we have used “food frequency” in the keywords

***50*** *: In all your text, it will be better to refer to preadolescents instead of children when you refer to your own study.*

**Response: **This term has been changed throughout the manuscript when it refers to the study.

***51*** *: You should say “included” instead of “involved” when you refer to a variable added in a model.* **Response: **This has been used accordingly in the manuscript

***52*** *: Figure 1: the dots in the lightest grey are not well visible. The saturation of the dots shows the total of preadolescents within those responses (the higher number of subjects, the more saturated the color in the chart).*

**Response: **We have adjusted the background of the figure

***53*** *Figure 3: here you also conducted multiple tests. So, you should not mention and comment p values above 0.05 and below or equal to 0.10.*

**Response: **The p-value was inserted to help in reading the figure and provide the statistical results.
